# Racial, ethnic and regional differences in the effect of sodium–glucose co-transporter 2 inhibitors and glucagon-like peptide 1 receptor agonists on cardiovascular and renal outcomes: a systematic review and meta-analysis of cardiovascular outcome trials

**DOI:** 10.1177/01410768231198442

**Published:** 2023-09-21

**Authors:** Setor K Kunutsor, Kamlesh Khunti, Samuel Seidu

**Affiliations:** Diabetes Research Centre, 4488 University of Leicester, Leicester General Hospital, Leicester LE5 4WP, UK

**Keywords:** GLP1-RA, SGLT2-I, type 2 diabetes, ethnicity, racial, randomised controlled trial

## Abstract

**Objectives:**

The cardiorenal protective effects of sodium–glucose co-transporter 2 inhibitors (SGLT2-Is) and glucagon-like peptide 1 receptor agonists (GLP1-RAs) across racial and ethnic groups are not well defined. By conducting a systematic review and meta-analysis of all randomised, placebo-controlled, cardiovascular disease (CVD) outcomes trials (CVOTs), we aimed to compare racial/ethnic as well as regional patterns in the effects of SGLT2-Is and GLP1-RAs on cardiovascular and renal outcomes in patients with type 2 diabetes (T2D).

**Design:**

Trials were identified from MEDLINE, Embase, the Cochrane Library, and search of bibliographies to 7 July 2023. Setting North America, South/Central America, Europe (Eastern and Western), Asia, Australia-New Zealand (Pacific), Asia/Pacific, and Africa.

**Setting:**

North America, South/Central America, Europe (Eastern and Western), Asia, Australia-New Zealand (Pacific), Asia/Pacific, and Africa.

**Participants:**

people with type 2 diabetes enrolled in cardiovascular outcome trials of SGLT2-Is and GLP1-RAs.

**Main outcome measures:**

Outcomes were (i) major adverse cardiovascular events (MACE), (ii) composite CVD death/heart failure (HF) hospitalization; (iii) composite renal outcome; and (iv) their components. Study-specific hazard ratios (HRs) with 95% confidence intervals (CIs) were pooled.

**Results:**

In total, 14 unique CVOTs (7 comparing SGLT2-Is vs placebo and 7 comparing GLP1-RAs vs placebo) were eligible. The proportion of participants enrolled in the trials ranged from 66.6-93.2% for White populations, 1.2-21.6% for Asian populations, 2.4-8.3% for Black populations and 0.9-23.1% for Other populations. The HR (95% CI) for MACE comparing SGLT2-Is vs placebo was 0.92 (0.86-0.98), 0.69 (0.53-0.92) and 0.70 (0.54-0.91) for White, Asian and Hispanic/Latino populations, respectively. Comparing GLP1-RAs vs placebo, the corresponding HR (95% CI) was 0.88 (0.80-0.97), 0.76 (0.63-0.93) and 0.82 (0.70-0.95), respectively. SGLT2-Is reduced the risk of all other cardiorenal outcomes in White and Asian populations, except for HF hospitalizations in Asians. No effects were observed in Black populations except for a reduced risk of HF hospitalizations by SGLT2-I. SGLT1-Is reduced the risk of composite CVD death/HF hospitalization in North America and Europe, whereas GLP1-RAs reduced the risk of MACE in Europe. GRADE certainty of evidence ranged from moderate to high.

**Conclusions:**

There appears to be substantial racial/ethnic differences in the cardiorenal effects of SGLT2-Is and GLP1-RAs in patients with T2D, with consistent benefits observed among White and Asian populations and consistent lack of benefits in Black populations. Whether the differences are due to issues with under-representation of Black populations and low statistical power or racial/ethnic variations in the pharmacokinetics, pharmacodynamics and safety of SGLT2-Is and GLP1-RAs need further investigation.

PROSPERO Registration: CRD42023401734

## Introduction

In 2021, about 537 million adults were living with diabetes, which is predicted to rise to 783 million by 2045.^1^ Diabetes is a leading cause of morbidity and mortality and associated with substantial socio-economic burden^2,3^; globally, there are about 1.5 million deaths attributable to diabetes annually.^4^ In 2019, diabetes (with type 2 diabetes [T2D] being the most common and accounting for around 90% of all cases of diabetes worldwide) was the fourth leading cause of disability globally; the number of disability-adjusted life years (DALYs) caused by diabetes was 66.3 million.^5^ Cardiovascular disease (CVD) is the leading cause of morbidity and mortality associated with T2D; renal disease due to diabetic nephropathy is also a common complication of T2D.^6^

Type 2 diabetes and its complications pose a global health threat.^7^ There are racial/ethnic differences in the prevalence of and risk factors for T2D and its microvascular and macrovascular complications as well as mortality.^8–10^ For instance, in the UK, the prevalence is higher among Asian, Black and minority ethnic groups than the White population^11^; ethnic minorities also carry a higher risk of developing diabetes at a younger age and at lower adiposity burden.^12^ In a number of comprehensive reviews of studies, mainly conducted in the USA and UK, ethnic minority populations (Blacks, Asians and Hispanics) were observed to be more likely than non-Hispanic Whites to have lower extremity amputations and develop retinopathy and nephropathy.^13,14^ Contributors to these variations include genetic background, health behaviours and biological, environmental, cultural and socioeconomic factors.^10^ Lifestyle modification, which includes weight loss, physical activity and healthy dietary patterns, remains one of the first-line strategies for the management of T2D. In addition to these, people with T2D need intensive glycaemic and risk factor control to reduce the risk of disease progression and complications.^15^ The quality of care including glycaemic control in T2D management has also been demonstrated to show racial/ethnic disparities.^16,17^ Older antihyperglycaemic agents, such as metformin (which until recently was the first-line drug therapy for patients with T2D), exert their microvascular and macrovascular benefits through their glycaemic control abilities. However, several newer therapeutic classes of antihyperglycemic agents introduced for T2D treatment now seem to have glucose-independent cardio-renal benefits. These agents include sodium–glucose co-transporter 2 inhibitors (SGLT2-Is) and glucagon-like peptide 1 receptor agonists (GLP1-RAs). The use of SGLT2-Is and GLP1-RAs, particularly, has increased because of their superior efficacy and lower risk of hypoglycaemia.^18,19^ Apart from their glucose-lowering effects, they have beneficial effects on blood pressure, weight control and renal function and significantly reduce the risk of adverse cardiovascular and kidney outcomes.^19–22^ For these reasons, recent guidelines preferentially recommend the initiation of these two classes of drugs.^23^

Minimising racial/ethnic variations in the cardiovascular and renal complications of T2D requires equal access to care and treatment and achieving optimal glycaemic and other risk factor (such as blood pressure and lipids) control in all populations. It is not known whether the efficacy of these newer agents is similar across different racial/ethnic groups. The pivotal cardiovascular outcome trials (CVOTs) that were the basis for the approval of these newer agents did not specifically take racial/ethnic variations into consideration during the design stage^19–22^; hence, their effects across different racial/ethnic groups are less well known. It will be clinically useful to summarise the existing evidence to provide more insight on the benefits and harms of these agents across different racial/ethnic groups, with particular focus on cardiovascular and renal outcomes. Our aim was to compare racial/ethnic as well as regional patterns in the cardiovascular and renal effectiveness and safety of SGLT2-Is and GLP1-RAs in patients with T2D, using a systematic meta-analysis of CVOTs of SGLT2-I and GLP1-RA.

## Methods

### Data sources and search strategy

This systematic meta-analysis was based on a pre-defined protocol and registered in the PROSPERO prospective register of systematic reviews (CRD42023401734). It was conducted in line with PRISMA guidelines (Supplementary Appendix 1). We conducted a database search of MEDLINE, Embase and the Cochrane Library from inception to 7 July 2023 with no restrictions on language. The computer-based searches combined free texts and MeSH terms related to T2D, GLP1RAs or SGLT2-Is, and cardiovascular outcomes. A randomised control trial (RCT) design search filter was employed. Details of the search algorithm are presented in Supplementary Appendix 2. Titles and abstracts of all retrieved citations were initially screened by one author (SKK) to assess the potential for inclusion. The screening was conducted using Rayyan, a free online bibliographic tool that helps expedite the screening via a process of semi-automation while incorporating high levels of usability.^24^ This was then followed by full text acquisition for detailed evaluation, which was independently conducted by two authors (SS and SKK). Disagreements regarding eligibility of an article/study were discussed and resolved by mutual agreement with involvement of all the authors. To acquire other articles that might have been missed by the search strategy, the reference lists of relevant studies and review articles were manually scanned and citing references were also checked in Web of Science.

### Study selection and eligibility criteria

Eligible studies were randomised, placebo-controlled CVOTs of SGLT2-Is or GLP1-RAs in patients with T2D, which have reported data on cardiovascular and/or renal outcomes by race/ethnicity and/or region of participants. The following studies were excluded: (i) studies that specifically enrolled only patients with known renal insufficiency or established renal parenchymal disease without T2D; (ii) non-CVOTs; (iii) studies that enrolled patients with type 1 diabetes; and (iv) studies that did not report any of the outcomes stratified by race/ethnicity.

### Data extraction

A predesigned data collection form, used in previous related reviews around the same topic,^25–27^ was adapted for data extraction. One experienced reviewer (SKK) initially extracted data from the eligible studies and a second experienced reviewer (SS) independently checked the extracted data for accuracy using the original articles. Any disagreements or inconsistencies were resolved by involvement of the other authors. Data were extracted on the following when available with stratification by race, ethnicity or region: study publication date, geographical location, patient characteristics (baseline age, sex, duration of T2D), study design characteristics (randomisation, allocation concealment, blinding, duration), intervention type and comparator groups, outcomes of interest and hazard ratios (HRs) with their 95% confidence intervals (CIs). A number of studies presented forest plots without the HRs and their 95% CIs; these estimates were extracted using Plotdigitizer, an online data extraction tool that allows users to extract data from images in numerical format (https://plotdigitizer.com/app).

### Outcomes

The pre-specified primary outcomes were defined as (i) major adverse cardiovascular events (MACE) or composite of death from cardiovascular causes, non-fatal myocardial infarction, or non-fatal stroke (composite cardiovascular outcome); (ii) composite outcome of CVD death/heart failure (HF) hospitalisation; (iii) composite outcome of end-stage kidney disease, doubling of creatinine level or death from renal causes (composite renal outcome); and (iv) all-cause mortality. Pre-specified secondary outcomes were (i) individual components of MACE or composite cardiovascular outcome; (ii) CVD death; (iii) HF hospitalisation; (iv) individual components of composite renal outcome; (v) other cardiovascular and renal outcomes; and (vi) adverse events (AEs).

### Risk of bias and certainty of evidence

The Cochrane Collaboration’s risk of bias tool was used to evaluate the risk of bias for each of the included trials.^28^ This tool evaluates seven possible sources of bias, which are random sequence generation, allocation concealment, blinding of participants and personnel, blinding of outcome assessment, incomplete outcome data, selective reporting and other bias. For each individual component, studies were classified into low, unclear and high risk of bias. We also assessed the certainty of the body of evidence for each outcome using the Grading of Recommendations Assessment, Development and Evaluation (GRADEpro) tool (https://gdt.gradepro.org), based on study limitations, inconsistency of effect, imprecision, indirectness and publication bias.^29^ The certainty of the evidence was rated at four levels: high, moderate, low and very low.

## Statistical analysis

Hazard ratios with 95% CIs were used as the summary measures of effect for all dichotomous outcomes. Random-effects models were used to combine HRs to minimise the effect of heterogeneity.^30^ Fixed-effects models were used in parallel analyses where appropriate. The extent of statistical heterogeneity across studies was quantified using standard chi-square tests and the I^2^ statistic.^31,32^ In our pre-specified protocol, we also planned to do the following: (i) explore for sources of heterogeneity using stratified analysis and random effects meta-regression; and (ii) assess for small study effects (e.g. publication bias) using formal tests such as Begg’s funnel plots^33^ and Egger’s regression symmetry test.^34^ However, these could not be done because each outcome stratified by race/ethnicity or region was based on pooled analysis of <10 studies. All tests were two-tailed and *p*-values of 0.05 or less were considered significant. All analyses were conducted using Stata version MP 16 (Stata Corp, College Station, TX, USA).

## Results

### Study identification and selection

**Figure 1.**
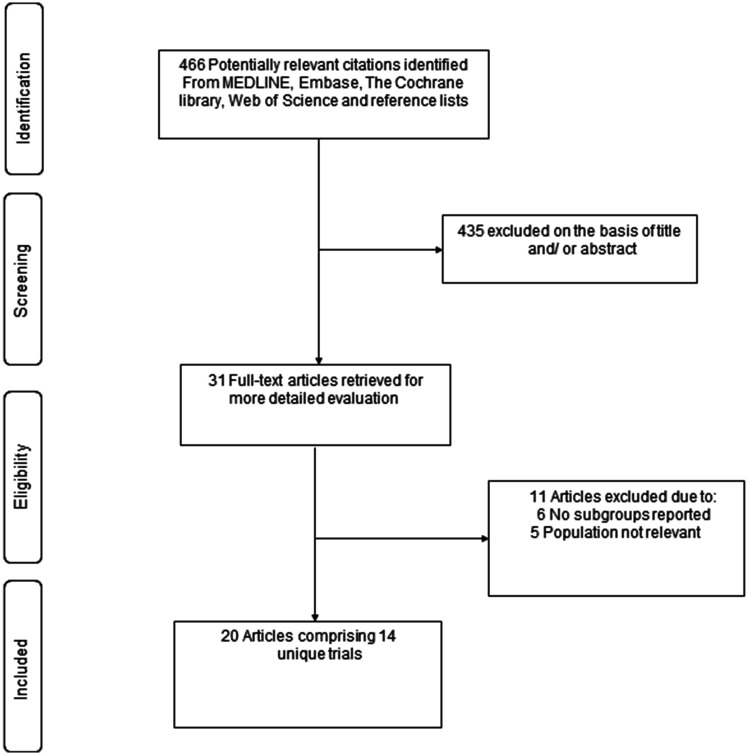
Selection of studies included in the meta-analysis.

Figure 1 shows the study selection process. The search of relevant databases and manual scanning of reference lists of relevant studies identified 466 potentially relevant citations. After the initial screening of titles and abstracts, 31 articles remained for full text evaluation. Following a detailed evaluation, 11 articles were excluded because (i) no subgroup results by race/ethnicity or region were reported (*n* = 6) and (ii) the populations recruited were not relevant to review question (*n* = 5). The remaining 20 articles met the inclusion criteria and were included in the review.^20–22,35–51^

### Study characteristics and risk of bias

The 20 articles comprised 14 unique double-blinded RCTs (seven involved the comparison of SGLT2-I with placebo and seven involved the comparison of GLP1-RA) that recruited patients with T2D and reported cardiovascular and/or renal outcomes by race, ethnicity or region (Table 1). Based on the available data, we stratified participants into the following groups and subgroups: (i) Race: White, Asian, Black and Other (e.g. American Indian, Alaska Native, Native Hawaiian, Multiracial); (ii) Ethnicity: Hispanic Latino and non-Hispanic Latino; and (iii) Region: North America, South/Central America, Europe (Eastern and Western), Asia, Australia-New Zealand (Pacific), Asia/Pacific and Africa. The proportion of participants enrolled in the trials ranged from 66.6% to 93.2% for White populations, 1.2% to 21.6% for Asian populations, 2.4% to 8.3% for Black populations and 0.9% to 23.1% for Other populations. In addition to T2D, patients had other co-existing conditions such as chronic kidney disease, high cardiovascular risk, atherosclerotic CVD or HF. All patients had been diagnosed with T2D (average duration ranged from 9.3 to 15.8 years) and were being managed on standard treatment therapies including background antihyperglycaemic and antihypertensive medications before inclusion into the trials. All the studies were conducted in multiple countries (ranging from 28 to 49 countries). The types of SGLT2-Is used included dapagliflozin, canagliflozin, empagliflozin, ertugliflozin and sotagliflozin and those for GLP1-RAs comprised albiglutide, dulaglutide, exenatide, liraglutide, lixisenatide, semaglutide and efpeglenatide. Non-compliance of trial medications was very low and ranged from 0.0% to 1.0% across trials that provided these data. The average age of participants ranged from 60 to 69 years. The mean/median duration of follow-up ranged from 9 months to 3.8 years. Using the Cochrane Collaboration tool, all trials demonstrated low risk of bias in all domains (Supplementary Appendix 3).

**Table 1.** Randomised, placebo-controlled, cardiovascular outcomes trials of sodium–glucose co-transporter 2 inhibitors and glucagon-like peptide 1 receptor agonists in type 2 diabetes.

Author, year of publication	Study name	Baseline population	Year of recruitment	Men, %	Mean/median age, years	Mean/median T2D duration, years	Location	Intervention and dose	Non-compliance (%)Intervention/Placebo	Mean/median follow-up duration, years
Zinman et al., 2015^20^; Wanner et al., 2016^47^; Fitchett et al., 2016^46^; Kaku et al., 2017^49^	EMPA-REG OUTCOME	T2D at high cardiovascular risk, eGFR ≥30	2010–2013	67.8	67.1	NR	590 sites in 42 countries	Empagliflozin 10/25 mg	0.6/0.6	3.1
Neal et al., 2017^21^; Perkovic et al., 2018^41^; Radholm et al., 2018^43^	CANVAS Program	T2D with high cardiovascular risk, eGFR > 30	2009, 2014	64.2	63.3	13.5	667 centres in 30 countries	Canagliflozin 100/300 mg	1.0/1.0	3.6
Cannon et al., 2020^37^; Cosentino et al., 2020^38^	VERTIS CV	T2D with atherosclerotic CVD	2013–2015/2016–2017	70.0	64.4	13.0	567 sites in 34 countries	Ertugliflozin 5/15 mg	0.1/0.0	3.5
Bhatt et al., 2021^35^	SCORED	T2D with CKD and additional cardiovascular risk	2017–2020	55.1	69.0	NR	750 sites in 44 countries	Sotagliflozin 200/400 mg	NR	1.3
Bhatt et al., 2021^36^	SOLOIST-WHF	T2D with worsening HF	2017–2020	66.3	69.0	NR	306 sites in 32 countries	Sotagliflozin 200/400 mg	NR	0.75
Wiviott et al., 2019^45^	DECLARE-TMI 58	T2D who had or were at risk for ASCVD	2013–2018	62.6	63.9	10.5	882 sites in 33 countries	Dapagliflozin 10 mg	NR	4.2
Perkovic et al., 2019^42^	CREDENCE	T2D and albuminuric chronic kidney disease	2014–2017	66.1	63.0	15.8	690 sites in 34 countries	Canagliflozin 100 mg	0.7/0.8	2.62
Pfeffer et al., 2015^44^	ELIXA	T2D and acute coronary syndrome	2010–2013	69.3	60.3	9.3	49 countries	Lixisenatide 10–20 mg	NR	2.1
Marso et al., 2016^22^	LEADER	T2D with at least 1 cardiovascular condition	2010–2012	60.7	64.6	12.8	410 sites in 32 countries	Liraglutide 1.8 mg	NR	3.8
Marso et al., 2016^40^	SUSTAIN-6	T2D	2013	60.7	64.6	13.9	230 sites in 20 countries	Semaglutide 0.5/1.0 mg	NR	2.1
Holman et al., 2017^39^	EXSCEL	T2D with or without CVD	2010–2015	62.0	62.0	12.0	687 sites in 35 countries	Exenatide 2 mg	NR	3.2
Hernandez et al., 2018^48^	Harmony outcomes	T2D and CVD	2015–2016	69.4	64.1	14.1	610 sites in 28 countries	Albiglutide 30–50 mg	NR	1.5
Gerstein et al., 2019^50^	REWIND	T2D with and without previous CVD	2011–2013	53.7	66.2	9.5	371 sites in 24 countries	Dulaglutide 1.5 mg	NR	5.4
Gerstein et al., 2021^51^	AMPLITUDE-O	T2D and history of CVD or kidney disease	2018–2019	67.0	64.5	15.4	344 sites in 28 countries	Efpeglenatide 4/6 mg	NR	1.8

ASCVD: atherosclerotic cardiovascular disease; CKD: chronic kidney disease; CVD: cardiovascular disease; eGFR: estimated glomerular filtration rate; HF: heart failure; L: liraglutide; NR: not reported; S: semaglutide; T2D: type 2 diabetes.

Study abbreviations – CANVAS: Canagliflozin Cardiovascular Assessment Study; CREDENCE: Canagliflozin and Renal Events in Diabetes with Established Nephropathy.

Clinical Evaluation – DECLARE-TMI 58: Dapagliflozin Effect on Cardiovascular Events–Thrombolysis in Myocardial Infarction 58; EMPA-REG OUTCOME: Empagliflozin Cardiovascular Outcome Event Trial in Type 2 Diabetes Mellitus Patients Removing Excess Glucose; ELIXA: Evaluation of Lixisenatide in Acute Coronary Syndrome; EXSCEL: Exenatide Study of Cardiovascular Event Lowering; LEADER: Liraglutide Effect and Action in Diabetes: Evaluation of Cardiovascular Outcome Results; REWIND: Researching Cardiovascular Events with a Weekly Incretin in Diabetes; SCORED: Effect of Sotagliflozin on Cardiovascular and Renal Events in Patients with Type 2 Diabetes and Moderate Renal Impairment Who Are at Cardiovascular Risk; SOLOIST-WHF: Effect of Sotagliflozin on Cardiovascular Events in Patients with Type 2 Diabetes Post Worsening Heart Failure; VERTIS CV: Evaluation of Ertugliflozin Efficacy and Safety Cardiovascular Outcomes.

### MACE

#### SGLT2-Is

**Figure 2.**
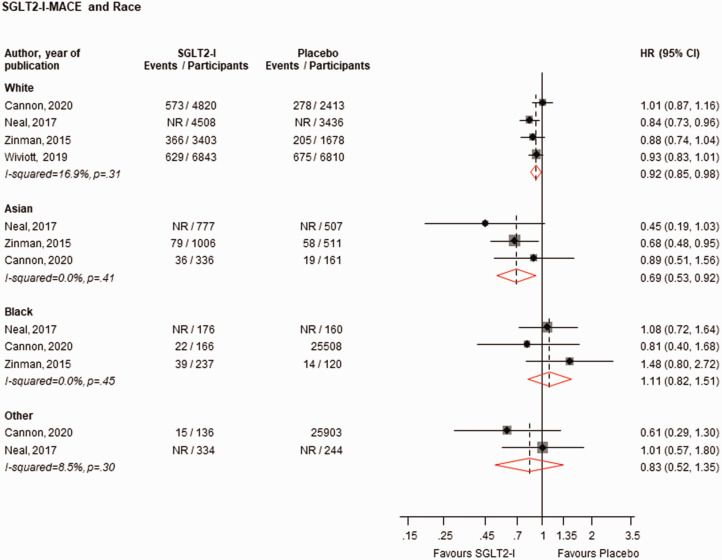
Risk for MACE comparing SGLT2-I with placebo by race.CI: confidence interval (bars); HR: hazard ratio; MACE: major adverse cardiovascular events; NR: not reported; SGLT2-I: sodium–glucose co-transporter 2 inhibitor.

**Figure 3.**
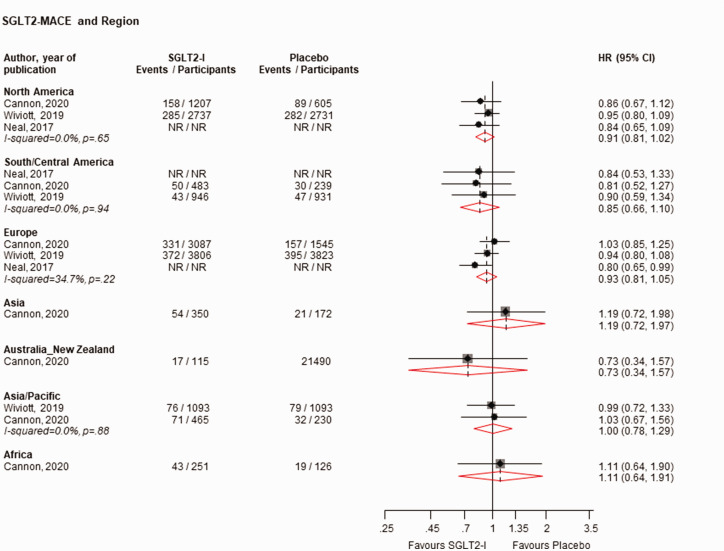
Risk for MACE comparing SGLT2-I with placebo by region.CI: confidence interval (bars); HR: hazard ratio; MACE: major adverse cardiovascular events; NR: not reported; SGLT2-I: sodium–glucose co-transporter 2 inhibitor.

When stratified by race, SGLT2-Is vs. placebo reduced the risk of MACE in White (*n* = 4 studies) and Asian (*n* = 3 studies) populations, with no strong evidence of an effect in Black (*n* = 3 studies) and Other (*n* = 2 studies) populations: HRs (95% CIs) of 0.92 (0.86–0.98), 0.69 (0.53–0.92), 1.11 (0.82–1.51) and 0.83 (0.52–1.35), respectively (Figure 2). There was no strong evidence of an effect on the risk of MACE when stratified by regions: HRs (95% CIs) of 0.91 (0.81–1.02), 0.85 (0.66–1.10), 0.93 (0.81–1.05), 1.19 (0.72–1.97), 0.73 (0.34–1.57), 1.00 (0.78–1.29) and 1.11 (0.64–1.91) for the regions of North America (*n* = 3 studies), South/Central America (*n* = 3 studies), Europe (*n* = 3 studies), Asia (*n* = 1 study), Pacific (*n* = 1 study), Asia/Pacific (*n* = 2 studies) and Africa (*n* = 1 study), respectively (Figure 3). When stratified by ethnicity, SGLT2-Is vs. placebo reduced the risk of MACE in Hispanic/Latinos (*n* = 2 studies), with no strong evidence of an effect in non-Hispanic/Latinos (*n* = 2 studies): HRs (95% CIs) of 0.70 (0.54–0.91) and 0.96 (0.86–1.07), respectively (Supplementary Appendix 4).

#### GLP1-RAs

**Figure 4.**
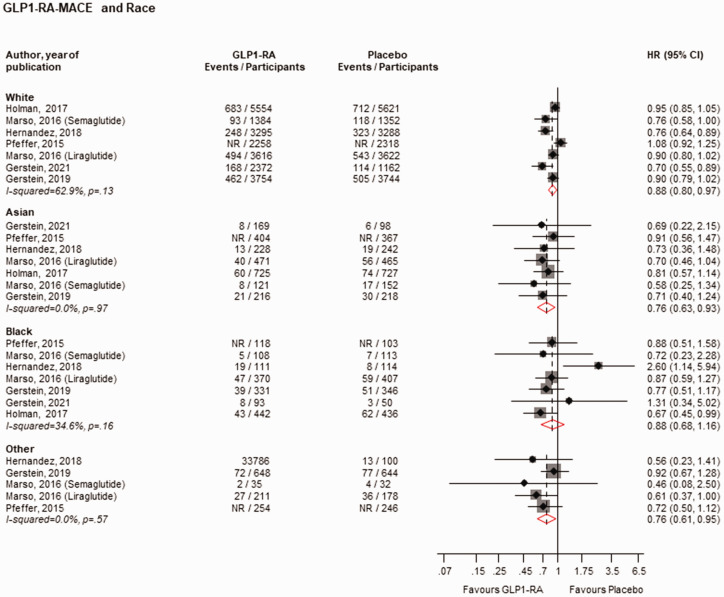
Risk for MACE comparing GLP1-RA with placebo by race.CI: confidence interval (bars); HR: hazard ratio; GLP1-RAs: glucagon-like peptide 1 receptor agonists; NR: not reported.

**Figure 5.**
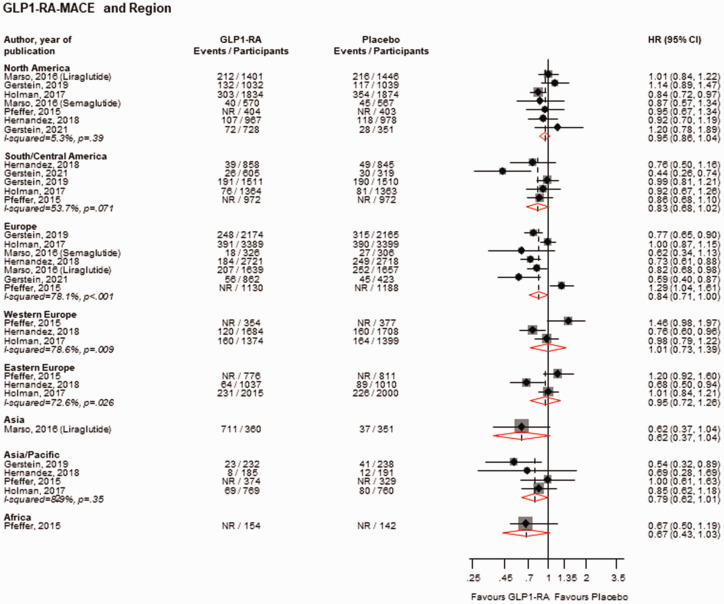
Risk for MACE comparing GLP1-RA with placebo by region.CI: confidence interval (bars); HR: hazard ratio; GLP1-RAs: glucagon-like peptide 1 receptor agonists; NR: not reported.

GLP1-RAs vs. placebo reduced the risk of MACE in White (*n* = 7 studies), Asian (*n* = 7 studies) and Other populations (*n* = 5 studies), but not in Black populations (*n* = 7 studies): HRs (95% CIs) of 0.88 (0.80–0.97), 0.76 (0.63–0.93), 0.76 (0.61–0.95) and 0.88 (0.68–1.16), respectively (Figure 4). Except for a reduction in the risk of MACE in the region of Europe (*n* = 7 studies), HR (95% CIs) of 0.84 (0.71–1.00), there was no strong evidence of an effect of GLP1-RAs vs. placebo on the risk of MACE across all the other regions: HRs (95% CIs) of 0.95 (0.86–1.04), 0.83 (0.68–1.02), 1.01 (0.73–1.39), 0.95 (0.72–1.26), 0.62 (0.37–1.04), 0.79 (0.62–1.01) and 0.67 (0.43–1.03) for North America (*n* = 7 studies), South/Central America (*n* = 5 studies), Western Europe (*n* = 3 studies), Eastern Europe (*n* = 3 studies), Asia (*n* = 1 study), Asia/Pacific (*n* = 4 studies) and Africa (*n* = 1 study), respectively (Figure 5). When stratified by ethnicity, GLP1-RAs vs. placebo reduced the risk of MACE in Hispanic/Latinos (*n* = 5 studies), with no strong evidence of an effect in non-Hispanic Latinos (*n* = 4 studies): HRs (95% CIs) of 0.82 (0.70–0.95) and 0.91 (0.78–1.06), respectively (Supplementary Appendix 5).

### Composite outcome of CVD death/HF hospitalisation

#### SGLT2-Is

SGLT2-Is vs. placebo reduced the risk of the composite outcome of CVD death/HF hospitalisation in White (*n* = 4 studies) and Asian (*n* = 2 studies), with no strong evidence of an effect in Black (*n* = 3 studies) and Other (*n* = 2 studies) populations: HRs (95% CIs) of 0.76 (0.65–0.90), 0.61 (0.42–0.89), 0.74 (0.49–1.13) and 0.77 (0.12–5.02), respectively (Supplementary Appendix 6). When stratified by regions, SGLT2-Is vs. placebo reduced the risk of the composite outcome of CVD death/HF hospitalisation for the regions of North America (*n* = 4 studies) and Europe (*n* = 5 studies), with no strong evidence of an effect for South/Central America (*n* = 4 studies), Asia (*n* = 1 study), Pacific (*n* = 1 study), Asia/Pacific (*n* = 2 studies) and Africa (*n* = 1 study): HRs (95% CIs) of 0.80 (0.69–0.92), 0.81 (0.72–0.91), 0.81 (0.64–1.01), 0.96 (0.50–1.86), 0.92 (0.31–2.74), 0.96 (0.67–1.38) and 1.00 (0.53–1.90), respectively (Supplementary Appendix 7). In single study results, there was no evidence of an effect on the composite outcome of CVD death/HF hospitalisation in Hispanic/Latinos and non-Hispanic/Latinos comparing SGLT2-Is vs. placebo: HRs (95% CIs) of 0.76 (0.50–1.15) and 0.90 (0.76–1.07), respectively (Supplementary Appendix 8).

### Composite outcome of end-stage kidney disease, doubling of creatinine level or death from renal causes

#### SGLT2-Is

The composite renal outcome stratified by region was reported by a single study. SGLT2-Is vs. placebo reduced the risk of the composite renal outcome for North America, with no evidence of an effect for Europe: HRs (95% CIs) of 0.37 (0.16–0.84) and 0.60 (0.29–1.24), respectively (Supplementary Appendix 9).

### All-cause mortality

#### SGLT2-Is

None of the eligible trials reported all-cause mortality outcomes by race/ethnicity or region. However, a pre-specified post hoc publication involving the EMPA-REG OUTCOME (Empagliflozin Cardiovascular Outcome Event Trial in Type 2 Diabetes Mellitus Patients Removing Excess Glucose) trial reported the effect of empagliflozin on cardiovascular outcomes in the subgroup of patients of Asian race.^49^ Empagliflozin vs. placebo marginally reduced all-cause mortality risk: HR (95% CI) of 0.64 (0.40–1.01).

### Cardiovascular death

#### SGLT2-Is

The outcome of CVD death by race was reported by one study. SGLT2-Is vs. placebo reduced the risk of CVD death in White and Asian populations but not in the Black population: HRs (95% CIs) of 0.64 (0.50–0.82), 0.44 (0.25–0.78) and 0.77 (0.33–1.79), respectively (Supplementary Appendix 10). Single study results showed that SGLT2-Is vs. placebo reduced the risk of CVD death in Hispanic/Latino and non-Hispanic/Latino: HRs (95% CIs) of 0.53 (0.32–0.88) and 0.64 (0.50–0.82), respectively (Supplementary Appendix 10).

### HF hospitalisation

#### SGLT2-Is

SGLT2-Is vs. placebo reduced the risk of HF hospitalisations in White (*n* = 2 studies) and Black (*n* = 2 studies), but not in Asian (*n* = 2 studies) and Other (*n* = 1 study) populations: HRs (95% CIs) of 0.72 (0.58–0.88), 0.43 (0.21–0.90), 0.70 (0.41–1.22) and 0.31 (0.09–1.09), respectively (Supplementary Appendix 11). In single study results, there was no evidence of an effect on HF hospitalisation in any of the regions evaluated, comparing SGLT2-Is vs. placebo (Supplementary Appendix 11).

### Other cardiovascular outcomes

#### SGLT2-Is

In the pre-specified post hoc subgroup analysis of patients of Asian race in the EMPA-REG OUTCOME trial,^49^ empagliflozin vs. placebo had no significant effect on fatal and non-fatal stroke, non-fatal stroke, fatal and non-fatal myocardial infarction and non-fatal myocardial infarction.

### Incident or worsening nephropathy

#### SGLT2-Is

Single study results showed that SGLT2-Is vs. placebo reduced the risk of incident or worsening nephropathy in White and Asian populations but not in the Black population: HRs (95% CIs) of 0.57 (0.48–0.67), 0.63 (0.49–0.81) and 1.01 (0.58–1.75), respectively (Supplementary Appendix 12).

### Composite outcome of end-stage kidney disease, 40% reduction in eGFR or death from renal causes

#### SGLT2-Is

The composite outcome of end-stage kidney disease, 40% reduction in eGFR or death from renal causes stratified by race and region, was reported by a single study. SGLT2-Is vs. placebo reduced the risk of this outcome in White and Other populations but not in the Black population: HRs (95% CIs) of 0.65 (0.48–0.88), 0.37 (0.14–0.99) and 0.68 (0.35–1.31), respectively (Supplementary Appendix 13). SGLT2-Is vs. placebo reduced the risk of this outcome for the regions of North America and Europe but not South/Central America: HRs (95% CIs) of 0.47 (0.29–0.76), 0.60 (0.40–0.90) and 0.35 (0.11–1.14), respectively (Supplementary Appendix 13).

### Composite outcome of end-stage kidney disease, doubling of creatinine level or CVD or renal death

#### SGLT2-Is

The composite renal and CVD outcome stratified by race and ethnicity was reported by a single study. SGLT2-Is vs. placebo reduced the risk of this outcome in White and Asian populations but not in Black and Other populations: HRs (95% CIs) of 0.70 (0.57–0.86), 0.66 (0.46–0.95), 0.83 (0.43–1.60) and 0.71 (0.43–1.18), respectively (Supplementary Appendix 14). SGLT2-Is vs. placebo reduced the risk of this composite outcome in both Hispanic/Latinos and non-Hispanic/Latinos: HRs (95% CIs) of 0.62 (0.47–0.81) and 0.74 (0.60–0.91), respectively (Supplementary Appendix 14).

### Adverse events

None of the trials reported AEs by race/ethnicity and/or region. However, a pre-specified post hoc publication involving the EMPA-REG OUTCOME trial conducted in patients of Asian race reported on AEs.^49^ The results show that the percentages of Asian patients who had any AEs, serious AEs or AEs leading to discontinuation of study intervention were similar in the empagliflozin and placebo groups.

### GRADE summary of findings

The GRADE working group recommends up to seven patient-important outcomes to be listed in the ‘summary of findings’ tables in systematic reviews.^29^ We selected outcomes for assessment based on their importance (primary outcomes) and how frequently they were reported by studies. GRADE certainty of the evidence ranged from moderate to high (Supplementary Appendix 15).

## Discussion

### Key findings

In a pooled analysis of 14 unique randomised, placebo-controlled CVOTs of SGLT2-Is and GLP1-RAs in patients with T2D, we examined racial/ethnic and regional patterns in the effects of these agents on various cardiovascular and renal outcomes. Our findings indicate that SGLT2-Is and GLP1-RAs reduced the risk of MACE in White, Asian and Hispanic/Latino populations. SGLT2-Is reduced the risk of all other cardiovascular and renal outcomes in White populations. Similarly, for Asian populations, SGLT2-I reduced the risk of all other cardiovascular and renal outcomes except for the composite outcome of CVD death/HF hospitalisation. No significant beneficial effects were observed in Black populations for both interventions and for all the evaluated outcomes, except for HF hospitalisations in relation to SGLT2-Is. However, some of the effect estimates were similar to those of other racial groups, but they did not reach statistical significance and were imprecise. We did not observe any regional differences in the effects of SGLT2-Is on MACE. However, GLP1-RAs reduced the risk of MACE specifically in the European region, while there was no strong evidence of effects in other regions. Furthermore, SGLT2-Is demonstrated a risk reduction in the composite outcome of CVD death/HF hospitalisation in North America and Europe, but no strong evidence of effects was found in other regions (Europe, South/Central America, Asia/Pacific and Africa). It is important to note that the findings regarding CVD death, incident or worsening nephropathy, and the composite renal and CVD outcome were based on single studies. The certainty of the overall evidence ranged from moderate to high.

### Comparison with previous studies

The current study cannot be directly compared with any previous studies on the topic, but a number of previous related reviews deserve mention and discussion. Cai et al. explored the racial/ethnic patterns in cardiovascular outcomes in trials of antidiabetic medications in people with T2D.^52^ A major strength of this review was the fact that they evaluated several antidiabetic medications in addition to SGLT2-Is and GLP1RAs, which included DPP-4 inhibitors, basal insulin, peroxisome proliferator-activated receptor antagonists and alpha glucosidase inhibitors. However, there were several relevant aspects that were not addressed. First, the review only focused on the outcome of MACE. Second, regional and some ethnic differences were not evaluated. Third, trials such as CANVAS (Canagliflozin Cardiovascular Assessment Study) and ELIXA (Evaluation of Lixisenatide in Acute Coronary Syndrome), which reported racial/ethnic and region-specific estimates, were not included in the review. Subsequent publications of the VERTIS CV (Evaluation of Ertugliflozin Efficacy and Safety Cardiovascular Outcomes) and EMPA-REG OUTCOME trials, which reported additional cardiovascular and renal outcomes by racial/ethnic and region, were also not included. Fourth, the review included trials such as the EMPEROR-Reduced (Empagliflozin Outcome Trial in Patients with Chronic Heart Failure and a Reduced Ejection Fraction) and DAPA-CKD (Dapagliflozin and Prevention of Adverse Outcomes in Chronic Kidney Disease), which were based in patients with and without T2D and did not specifically report racial/ethnic and region-specific estimates for T2D. Qiu et al. evaluated the effects of SGLT2-Is and GLP1-RAs on only the outcome of MACE by race, ethnicity and region based on a pooled analysis of 11 CVOTs; consistent with our results, they showed no evidence of an effect of SGLT2-Is and GLP1-RAs on the risk of MACE in Black populations.^53^ Another recent meta-analysis of CVOTs by Lee et al. only compared the cardiovascular effects of SGLT2-Is and GLP1-RAs in Asian versus White populations with and without T2D.^54^ The results suggested differential treatment effects of SGLT2-Is and GLP-1RAs by race (White and Asian populations), with greater benefits of both classes of drugs in Asians.

Compared to the study by Cai et al.,^52^ we only focused on SGLT2-Is and GLP1-RAs because among the newer therapeutic agents for the treatment of T2D, only members of these two classes have been shown to significantly reduce the risk of major cardiovascular events such as MACE^19–22^; they have also been the focus of guideline recommendations.^23^ Three of the DPP-4Is (saxagliptin, alogliptin and sitagliptin) have been demonstrated to have no effect on MACE^55–57^; furthermore, the SAVOR-TIMI 53 (Saxagliptin Assessment of Vascular Outcomes Recorded in Patients with Diabetes Mellitus-Thrombolysis in Myocardial Infarction 53) trial showed an increased risk of HF hospitalisations,^55^ findings that were unexpected and very concerning. We evaluated a comprehensive panel of cardiovascular and renal outcomes in addition to safety outcomes as reported by the trials. In addition to race data, we also evaluated ethnic (Hispanic/Latino vs. non-Hispanic/Latino) and regional patterns in outcomes, which were reported by the majority of trials included. Finally, we only included trials that recruited patients with only T2D; hence, our results can be generalised to this specific population group.

### Potential explanation of findings

Over the last decade, several landmark CVOTs have consistently demonstrated the cardiovascular and renal protective effects of SGLT2-Is and GLP1-RAs.^19–22^ SGLT2-Is work by targeting renal tubular glucose reabsorption, thereby exerting glucose-lowering effects through increased urinary glucose excretion.^58^ GLP1-RAs exert their glucose-lowering effects via glucose-dependent increase in insulin secretion, inhibition of glucagon secretion, reduction in postprandial glucose excretion and increase in satiety.^59^ It has been reported that both classes of drugs may exert their cardiorenal protective effects independently of glucose control via their individual pleiotropic properties.^59^

The main findings showed consistent reductions in risk of adverse cardiovascular and renal outcomes for White and Asian populations, with no evidence of beneficial effects for Black and Other populations. Though some of the effect estimates were similar for these populations, they were imprecise and did not reach statistical significance for Black populations. These findings may suggest inadequate power to demonstrate any effects, which is reflected in the low sample size and event rates for these populations. Indeed, these findings were based on results from subgroup analyses and the trials were rather designed and powered for the overall populations recruited. Furthermore, given the consistent nature of the significant lack of beneficial effects across the majority of outcomes for Black populations, other factors, apart from power issues, may also be at play here. It is well known that Black populations have a higher prevalence of major cardiovascular risk factors such as hypertension, dyslipidaemia, smoking, physical inactivity and other co-morbidities compared with White populations.^60^ This suggests that Black populations already have a higher baseline risk for developing adverse cardiovascular and renal events. The majority of these factors require lifestyle modifications and other interventions and cannot be controlled by the trial interventions. None of the included trials reported a breakdown of baseline characteristics according to race/ethnicity, so it is difficult to know the baseline risk profile of the various groups. Differences in medication compliance and diabetes control across the various ethnic and racial groups in the trials might also account for these findings; however, none of the trials reported on these data according to race/ethnicity. Finally, there may be racial/ethnic differences in the pharmacokinetics, pharmacodynamics and safety of SGLT2-Is and GLP1-RAs; hence, their effects may differ. For example, it is well known that there are differences in the degree and rate of response of Black and White populations to various antihypertensive medications. For instance, diuretics and calcium channel blockers (CCBs) are more effective in older Black patients than beta blockers or ACE inhibitors, whereas White patients, especially younger individuals, appear to respond somewhat better to beta blockers and ACE inhibitors than to diuretics or CCBs.^61^ In a review to determine the efficacy of newer treatments in White and Asian populations with diabetes, the findings suggested that the glucose-lowering efficacy of SGLT2-Is, and to a lesser extent DPP-4Is, was greater in Asian populations compared with White populations; no difference was observed for GLP1-RAs.^62^ However, in a 24-week placebo-controlled, double-blind, parallel-group RCT to study the effects of empagliflozin in Black patients with T2D and hypertension, empagliflozin reduced glycated haemoglobin, body weight and blood pressure and it was well tolerated during the study period.^63^ It is not known if these beneficial changes will translate to reductions in adverse cardiovascular and renal outcomes as patients were not followed up for these outcomes. The mechanistic pathways underlying the cardiorenal protective effects of SGLT2-Is and GLP1RAs and their potential racial/ethnic differences warrant investigation.

### Implications of findings

Given the well-documented evidence that Black and other ethnic minority populations are more likely to develop T2D and at a younger age, have lower rates of risk factor control and have higher lifetime risk of developing microvascular and macrovascular complications,^12–14,64^ the consistent lack of benefits observed among Black populations is concerning. The global burden of T2D has increased substantially in recent decades and will continue to soar in the next few decades; this increase will be accompanied by a proportionate increase in morbidity and deaths attributable to the disease. This trend is likely to disproportionately impact on populations at high risk of developing the disease and with very little access to care and effective treatments. Newer and more effective therapeutic agents have been developed for the management of T2D, and guidelines suggest that they may be appropriate for all populations; however, the evidence seems to suggest that the beneficial effects are not consistent across all populations. As discussed, though there are many factors that could have contributed to the lack of evidence of beneficial effects for Black and other non-White populations, low statistical power due to small sample sizes of these populations may partly be responsible for the current findings. It is quite clear from the current data that some racial/ethnic groups such as Black populations were underrepresented in all the included trials. Data suggest that ethnic minorities are under-represented in CVOTs of T2D and that these trials may be characterised by recruitment bias.^65,66^ Furthermore, reporting of baseline and trial characteristics and outcome data according to race/ethnicity is far from optimal in these trials. To echo Khunti et al.,^66^ efforts should be made to improve reporting of race/ethnicity and improve diversity in trial recruitment. It is a fact that populations that are often under-represented in these trials are the ones who are more likely to receive the interventions being evaluated. Given that regulatory bodies now require the conduct of separate efficacy and safety studies for paediatric populations, we recommend that regulatory bodies propose a diversity recruitment threshold when such trials are being conducted to improve diversity. The long-term cardiorenal efficacy and safety of these antihyperglycaemic agents deserve specific investigation in these underrepresented populations to ascertain if the current findings are related to statistical power or to the specific mechanisms underlying the cardiorenal protective effects of these agents. In the absence of robust efficacy and safety data among different ethnic/racial groups, the use of these newer agents needs to be individualised based on patients’ unique circumstances.

## Strengths and limitations

In addition to the several strengths mentioned above, we identified major evidence gaps, which is the general lack of an attempt by major CVOT in T2D to ensure racial/ethnic balance during recruitment and also report outcomes by race/ethnicity. To the best of our knowledge, we included all known CVOTs in T2D that had reported outcomes by race/ethnicity and/or region. For trials that presented forest plots but did not report actual HRs with their 95% CIs, we were able to extract these estimates using an appropriate tool. We also included additional publications of these trials that had reported relevant data for inclusion in our review. Finally, we were also able to quantitatively summarise most of the available data, assess the risk of bias in individual trials and rate the certainty of the outcome evidence using validated tools. Several limitations were inherent but deserve consideration. First, though we pre-specified a long list of outcomes, most studies only reported a breakdown of the primary outcome (MACE). Some of our outcomes were based on results from single studies. Furthermore, none of the studies reported a breakdown by AEs, which was also a pre-specified outcome for our review. Adverse events were only reported by the pre-specified post hoc publication involving the EMPA-REG OUTCOME trial in the subgroup of patients of Asian race.^49^ Second, the definitions of race/ethnicity somewhat varied across trials, which precluded consistent harmonisation of the data. We acknowledge the fact that classification of race/ethnicity is a complex concept and it can also be challenging to collect such data.^67^ Third, our pooled data are based on results from subgroup analysis, which may be prone to bias. Fourth, the limited number of trials (*n* < 10) for all outcomes precluded accurate estimation of between-study heterogeneity, exploration of sources of heterogeneity and assessment of small study effects. Fourth, though heterogeneity estimates generally suggested low levels of heterogeneity across trials for most outcomes, pooling is still controversial given the differences in baseline characteristics, treatment types, duration of treatment and follow-up and reporting in outcome definitions. Access to individual-level data may help address issues related to race/ethnicity coding and ascertain if the current results are due to true differences or low statistical power.

## Conclusions

Pooled evidence from CVOTs of T2D demonstrates substantial racial/ethnic differences in the cardiorenal effects of SGLT2-Is and GLP1-RAs in patients with T2D, with most benefits observed among White and Asian populations. There is consistent lack of evidence of beneficial effects in Black populations.

Whether the differences are due to issues with under-representation of Black populations and low statistical power or racial/ethnic variations in the pharmacokinetics, pharmacodynamics and safety of SGLT2-Is and GLP1-RAs need further investigation.

## Supplemental Material

sj-pdf-1-jrs-10.1177_01410768231198442 - Supplemental material for Racial, ethnic and regional differences in the effect of sodium–glucose co-transporter 2 inhibitors and glucagon-like peptide 1 receptor agonists on cardiovascular and renal outcomes: a systematic review and meta-analysis of cardiovascular outcome trials Supplemental material, sj-pdf-1-jrs-10.1177_01410768231198442 for Racial, ethnic and regional differences in the effect of sodium–glucose co-transporter 2 inhibitors and glucagon-like peptide 1 receptor agonists on cardiovascular and renal outcomes: a systematic review and meta-analysis of cardiovascular outcome trials by Setor K Kunutsor, Kamlesh Khunti and Samuel Seidu in Journal of the Royal Society of Medicine

sj-pdf-2-jrs-10.1177_01410768231198442 - Supplemental material for Racial, ethnic and regional differences in the effect of sodium–glucose co-transporter 2 inhibitors and glucagon-like peptide 1 receptor agonists on cardiovascular and renal outcomes: a systematic review and meta-analysis of cardiovascular outcome trials Supplemental material, sj-pdf-2-jrs-10.1177_01410768231198442 for Racial, ethnic and regional differences in the effect of sodium–glucose co-transporter 2 inhibitors and glucagon-like peptide 1 receptor agonists on cardiovascular and renal outcomes: a systematic review and meta-analysis of cardiovascular outcome trials by Setor K Kunutsor, Kamlesh Khunti and Samuel Seidu in Journal of the Royal Society of Medicine

## Declarations

### Guarantor

SKK, KK, SS

### Data sharing

The corresponding author had full access to all the data in the study and takes responsibility for the integrity of the data and the accuracy of the data analysis. This study is based on data from published articles. The supplementary material are all included in this submission.

### Provenance

Not commisioned; peer reviewed by David Goldsmith and Julie Morris.
