# Supplementary material for: Racial, ethnic and regional differences in the effect of sodium–glucose co-transporter 2 inhibitors and glucagon-like peptide 1 receptor agonists on cardiovascular and renal outcomes: a systematic review and meta-analysis of cardiovascular outcome trials
Source: J R Soc Med. 2023 Sep 21;117(8):267–83. doi: 10.1177/01410768231198442 (PMC11450921; doi:10.1177/01410768231198442)
Supplement: sj-pdf-2-jrs-10.1177_01410768231198442 - Supplemental material for Racial, ethnic and regional differences in the effect of sodium–glucose co-transporter 2 inhibitors and glucagon-like peptide 1 receptor agonists on cardiovascular and renal outcomes: a systematic review and meta-analysis of cardiova [file sj-pdf-2-jrs-10.1177_01410768231198442.pdf]

SUPPLEMENTARY MATERIAL

**Appendix 1**	PRISMA checklist
**Appendix 2**	MEDLINE literature search strategy
**Appendix 3**	Assessment of risk of bias
**Appendix 4**	Risk for MACE comparing SGLT2-Is with placebo by ethnicity
**Appendix 5**	Risk for MACE comparing GLP1-RAs with placebo by ethnicity
**Appendix 6**	Risk for the composite outcome of CVD death or HF hospitalization comparing SGLT2-Is with placebo by race
**Appendix 7**	Risk for the composite outcome of CVD death or HF hospitalization comparing SGLT2-Is with placebo by region
**Appendix 8**	Risk for the composite outcome of CVD death or HF hospitalization comparing SGLT2-Is with placebo by ethnicity
**Appendix 9**	Risk for the composite renal outcome comparing SGLT2-Is with placebo by region
**Appendix 10**	Risk for CVD death comparing SGLT2-Is with placebo by race and ethnicity
**Appendix 11**	Risk for HF hospitalization comparing SGLT2-Is with placebo by race and region
**Appendix 12**	Risk for incident or worsening nephropathy comparing SGLT2-Is with placebo by race
**Appendix 13**	Risk for the composite outcome of end-stage kidney disease, 40% reduction in eGFR or death from renal causes comparing SGLT2-Is with placebo by race and region
**Appendix 14**	Risk for the composite outcome of end-stage kidney disease, doubling of creatinine level or CVD or renal death comparing SGLT2-Is with placebo by race and ethnicity
**Appendix 15**	GRADE summary of findings

Appendix 1: PRISMA checklist

Section/topic	Item No	Checklist item	Reported on page No
**Title**			
Title	1	Identify the report as a systematic review, meta-analysis, or both	1
**Abstract**			
Structured summary	2	Provide a structured summary including, as applicable, background, objectives, data sources, study eligibility criteria, participants, interventions, study appraisal and synthesis methods, results, limitations, conclusions and implications of key findings, systematic review registration number	2
**Introduction**			
Rationale	3	Describe the rationale for the review in the context of what is already known	Introduction
Objectives	4	Provide an explicit statement of questions being addressed with reference to participants, interventions, comparisons, outcomes, and study design (PICOS)	Introduction
**Methods**			
Protocol and registration	5	Indicate if a review protocol exists, if and where it can be accessed (such as web address), and, if available, provide registration information including registration number	Methods
Eligibility criteria	6	Specify study characteristics (such as PICOS, length of follow-up) and report characteristics (such as years considered, language, publication status) used as criteria for eligibility, giving rationale	Methods
Information sources	7	Describe all information sources (such as databases with dates of coverage, contact with study authors to identify additional studies) in the search and date last searched	Methods
Search	8	Present full electronic search strategy for at least one database, including any limits used, such that it could be repeated	Appendix 2
Study selection	9	State the process for selecting studies (that is, screening, eligibility, included in systematic review, and, if applicable, included in the meta-analysis)	Methods
Data collection process	10	Describe method of data extraction from reports (such as piloted forms, independently, in duplicate) and any processes for obtaining and confirming data from investigators	Methods
Data items	11	List and define all variables for which data were sought (such as PICOS, funding sources) and any assumptions and simplifications made	Methods
Risk of bias in individual studies	12	Describe methods used for assessing risk of bias of individual studies (including specification of whether this was done at the study or outcome level), and how this information is to be used in any data synthesis	Methods
Summary measures	13	State the principal summary measures (such as risk ratio, difference in means).	Methods
Synthesis of results	14	Describe the methods of handling data and combining results of studies, if done, including measures of consistency (such as I^2 statistic) for each meta-analysis	Methods
Risk of bias across studies	15	Specify any assessment of risk of bias that may affect the cumulative evidence (such as publication bias, selective reporting within studies)	Methods
Additional analyses	16	Describe methods of additional analyses (such as sensitivity or subgroup analyses, meta-regression), if done, indicating which were pre-specified	Methods
**Results**			
Study selection	17	Give numbers of studies screened, assessed for eligibility, and included in the review, with reasons for exclusions at each stage, ideally with a flow diagram	Results, Figure 1
Study characteristics	18	For each study, present characteristics for which data were extracted (such as study size, PICOS, follow-up period) and provide the citations	Results, Table 1
Risk of bias within studies	19	Present data on risk of bias of each study and, if available, any outcome-level assessment (see item 12).	Results, Appendix 3
Results of individual studies	20	For all outcomes considered (benefits or harms), present for each study (a) simple summary data for each intervention group and (b) effect estimates and confidence intervals, ideally with a forest plot	
Synthesis of results	21	Present results of each meta-analysis done, including confidence intervals and measures of consistency	Results, Figures 2-5; Appendices 4-14
Risk of bias across studies	22	Present results of any assessment of risk of bias across studies (see item 15)	Not applicable
Additional analysis	23	Give results of additional analyses, if done (such as sensitivity or subgroup analyses, meta-regression) (see item 16)	Results
**Discussion**			
Summary of evidence	24	Summarise the main findings including the strength of evidence for each main outcome; consider their relevance to key groups (such as health care providers, users, and policy makers)	Discussion
Limitations	25	Discuss limitations at study and outcome level (such as risk of bias), and at review level (such as incomplete retrieval of identified research, reporting bias)	Discussion
Conclusions	26	Provide a general interpretation of the results in the context of other evidence, and implications for future research	Discussion
**Funding**			
Funding	27	Describe sources of funding for the systematic review and other support (such as supply of data) and role of funders for the systematic review	After Discussion

Appendix 2: MEDLINE literature search strategy

1 dapagliflozin.mp. (2408)

2 exp Canagliflozin/ (951)

3 empagliflozin.mp. (2515)

4 ertugliflozin.mp. (227)

5 sotagliflozin.mp. (151)

6 albiglutide.mp. (228)

7 exp Exenatide/ (2846)

8 exp Liraglutide/ (2408)

9 lixisenatide.mp. (545)

10 semaglutide.mp. (995)

11 type 2 diabetes.mp. or exp Diabetes Mellitus, Type 2/ (220674)

12 exp Mortality/ (421925)

13 exp Myocardial Infarction/ or major adverse cardiovascular events.mp. or exp Acute Coronary Syndrome/ or exp Cardiovascular Diseases/ (2688561)

14 exp Heart Failure/ (144472)

15 cardiovascular outcome.mp. (2724)

16 ("clinical trial" or "clinical trial, phase i" or "clinical trial, phase ii" or clinical trial, phase iii or clinical trial, phase iv or controlled clinical trial or "multicenter study" or "randomized controlled trial").pt. or double-blind method/ or clinical trials as topic/ or clinical trials, phase i as topic/ or clinical trials, phase ii as topic/ or clinical trials, phase iii as topic/ or clinical trials, phase iv as topic/ or controlled clinical trials as topic/ or randomized controlled trials as topic/ or early termination of clinical trials as topic/ or multicenter studies as topic/ or ((randomi?ed adj7 trial*) or (controlled adj3 trial*) or (clinical adj2 trial*) or ((single or doubl* or tripl* or treb*) and (blind* or mask*))).ti,ab,kw. or ("4 arm" or "four arm").ti,ab,kw. (1898617)

17 1 or 2 or 3 or 4 or 5 or 6 or 7 or 8 or 9 or 10 (11070)

18 12 or 13 or 14 or 15 (3014236)

19 11 and 16 and 17 and 18 (885)

20 limit 19 to (humans and "all adult (19 plus years)") (396)

****

Each part was specifically translated for searching alternative databases.

Appendix 3: Assessment of risk of bias

	Randon sequence generation	Allocation concealment	Blinding of participants & personnel	Blinding of outcome assessments	Incomplete outcome data	Selective reporting	Other bias
**CANVAS Program**	+	+	+	+	+	+	+
**CREDENCE**	+	+	+	+	+	+	+
**DECLARE-TMI 58**	+	+	+	+	+	+	+
**EMPA-REG OUTCOME**	+	+	+	+	+	+	+
**SCORED**	+	+	+	+	+	+	+
**SOLOIST-WHF**	+	+	+	+	+	+	+
**VERTIS CV**	+	+	+	+	+	+	+
**ELIXA**	+	+	+	+	+	+	+
**EXSCEL**	+	+	+	+	+	+	+
**Harmony Outcomes**	+	+	+	+	+	+	+
**Leader**	+	+	+	+	+	+	+
**REWIND**	+	+	+	+	+	+	+
**SUSTAIN-6**	+	+	+	+	+	+	+
**AMPLITUDE-O**	+	+	+	+	+	+	+

+	Low risk of bias
?	Unclear risk of bias
-	High risk of bias

Study Abbreviations: CANVAS, Canagliflozin Cardiovascular Assessment Study; CREDENCE, Canagliflozin and Renal Events in Diabetes with Established Nephropathy Clinical Evaluation; DECLARE-TMI 58, Dapagliflozin Effect on Cardiovascular Events–Thrombolysis in Myocardial Infarction 58; EMPA-REG OUTCOME, Empagliflozin Cardiovascular Outcome Event Trial in Type 2 Diabetes Mellitus Patients Removing Excess Glucose; ELIXA, Evaluation of Lixisenatide in Acute Coronary Syndrome; EXSCEL, Exenatide Study of Cardiovascular Event Lowering; LEADER, Liraglutide Effect and Action in Diabetes: Evaluation of Cardiovascular Outcome Results; REWIND, Researching Cardiovascular Events with a Weekly Incretin in Diabetes; SCORED, Effect of Sotagliflozin on Cardiovascular and Renal Events in Patients with Type 2 Diabetes and Moderate Renal Impairment Who Are at Cardiovascular Risk; SOLOIST-WHF, Effect of Sotagliflozin on Cardiovascular Events in Patients with Type 2 Diabetes Post Worsening Heart Failure; VERTIS CV, Evaluation of Ertugliflozin Efficacy and Safety Cardiovascular Outcomes

Appendix 4: Risk for MACE comparing SGLT2-I with placebo, by ethnicity

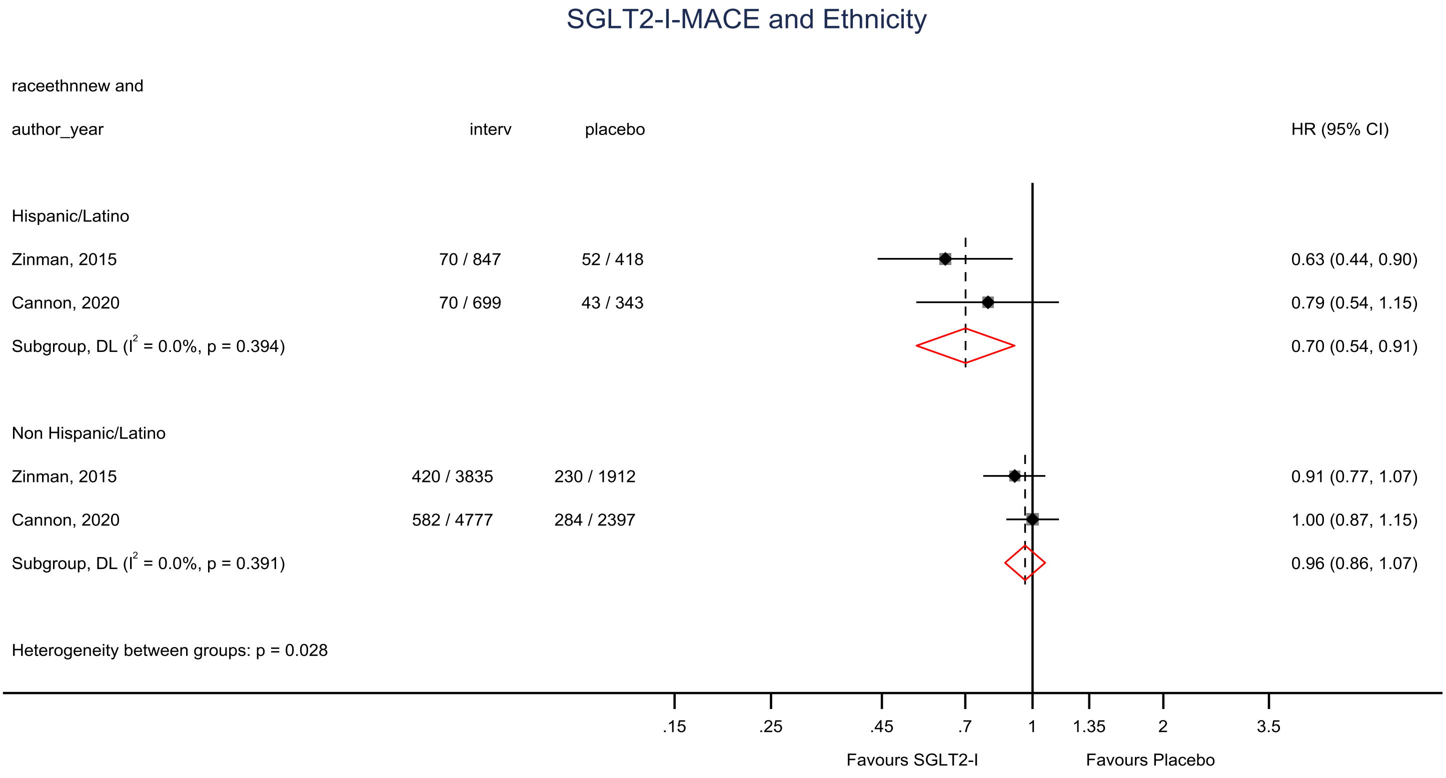

CI, confidence interval (bars); HR, hazard ratio; MACE, major adverse cardiovascular events; SGLT2-I, sodium–glucose co-transporter 2 inhibitor

Appendix 5: Risk for MACE comparing GLP1-RA with placebo, by ethnicity

GLP1-RA-MACE and Ethnicity

Author, year of publication	GLP1-RA Events / Participants	Placebo Events / Participants	HR (95% CI)
**Hispanic/Latino**			
Pfeffer, 2015	NR / NR	NR / NR	0.89 (0.69, 1.15)
Marso, 2016 (Semaglutide)	13 / 256	19 / 254	0.67 (0.33, 1.36)
Marso, 2016 (Liraglutide)	68 / 580	86 / 554	0.74 (0.54, 1.02)
Holman, 2017	47 / 577	49 / 557	0.93 (0.62, 1.38)
Hernandez, 2018	51 / 1005	65 / 988	0.74 (0.52, 1.07)
I-squared=0.0%, p=.77			0.82 (0.70, 0.95)
**Non Hispanic/Latino**			
Pfeffer, 2015	NR / NR	NR / NR	1.06 (0.91, 1.25)
Holman, 2017	6 / 56	7 / 52	0.86 (0.29, 2.54)
Marso, 2016 (Semaglutide)	95 / 1392	127 / 1395	0.74 (0.57, 0.96)
Marso, 2016 (Liraglutide)	540 / 4088	608 / 4118	0.89 (0.79, 1.00)
I-squared=50.5%, p=.11			0.91 (0.78, 1.06)

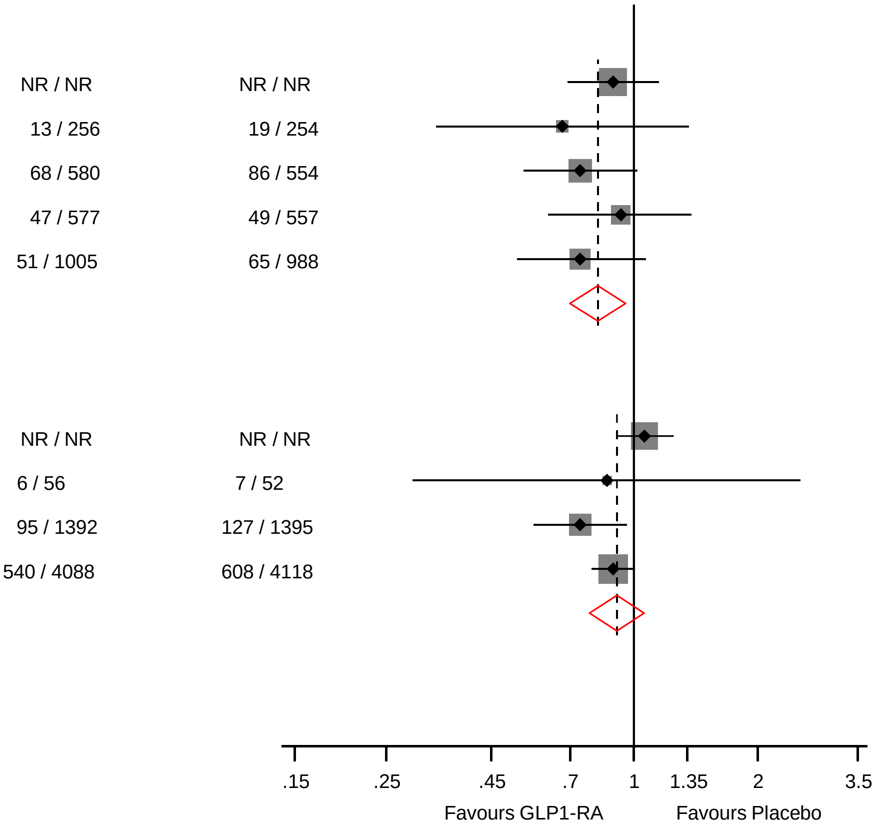

CI, confidence interval (bars); GLP1-RA, glucagon-like peptide 1 receptor agonist; HR, hazard ratio; MACE, major adverse cardiovascular events; NR, not reported

Appendix 6: Risk for the composite outcome of CVD death or HF hospitalization comparing SGLT2-I with placebo, by ethnicity

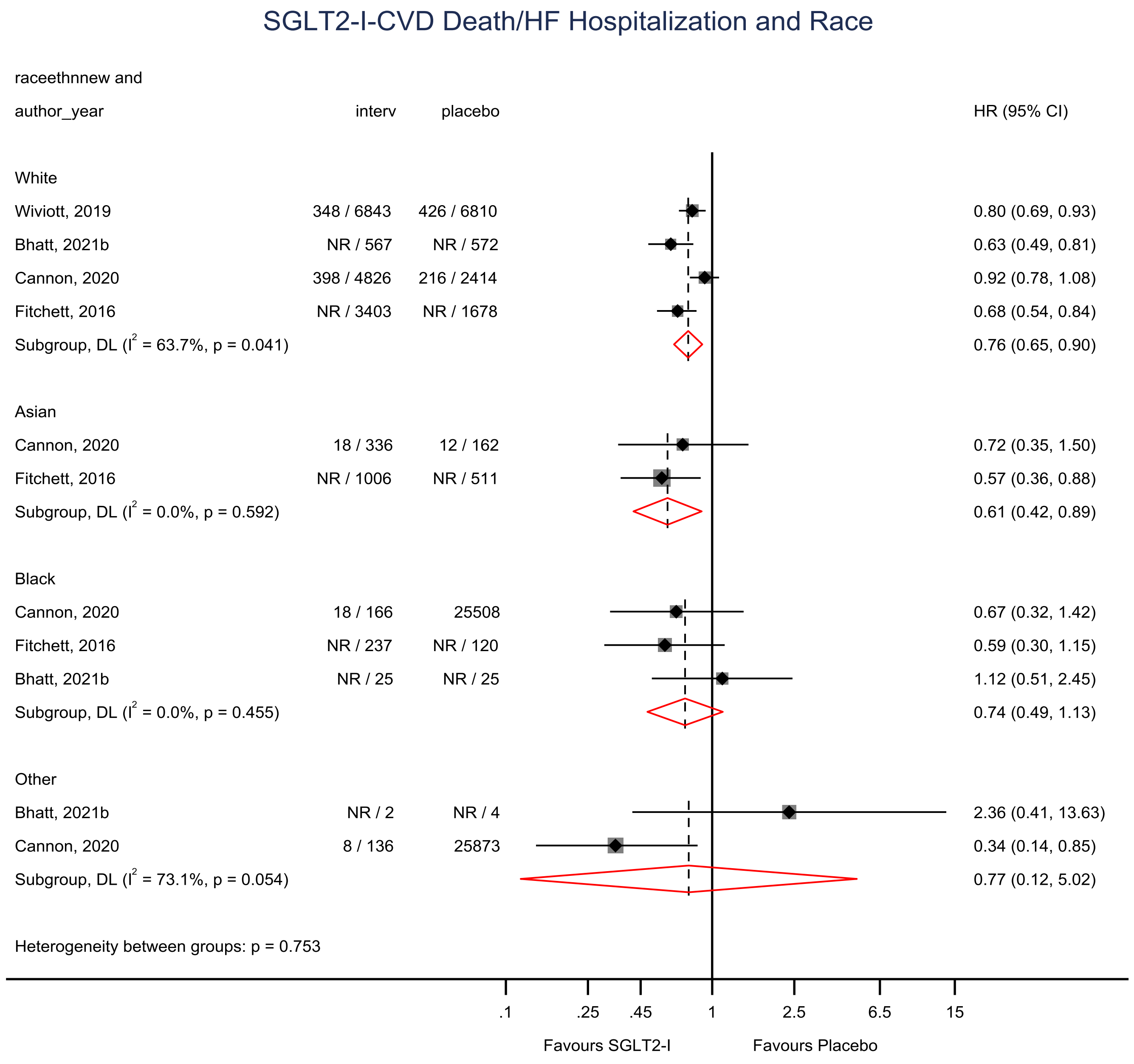

CI, confidence interval (bars); CVD, cardiovascular disease; HF, heart failure; HR, hazard ratio; MACE, major adverse cardiovascular events; NR, not reported; SGLT2-I, sodium–glucose co-transporter 2 inhibitor

Appendix 7: Risk for the composite outcome of CVD death or HF hospitalization comparing SGLT2-I with placebo by region

SGLT2-I-CVD Death/HF Hospitalization and Region

Author, year of publication	SGLT2-I Events / Participants	Placebo Events / Participants	HR (95% CI)
**North America**			
Wiviott, 2019	138 / 2737	185 / 2731	0.73 (0.59, 0.90)
Radholm, 2018	NR / NR	NR / NR	0.87 (0.63, 1.20)
Bhatt, 2021a	NR / 746	NR / 747	0.92 (0.60, 1.40)
Cannon, 2020	118 / 1208	71 / 605	0.82 (0.61, 1.10)
I-squared=0.0%, p=.70			0.80 (0.69, 0.92)
**South/Central America**			
Wiviott, 2019	32 / 946	37 / 931	0.85 (0.51, 1.34)
Bhatt, 2021a	NR / 1586	NR / 1586	0.77 (0.54, 1.10)
Cannon, 2020	39 / 484	24 / 239	0.80 (0.48, 1.32)
Radholm, 2018	NR / NR	NR / NR	0.84 (0.50, 1.43)
I-squared=0.0%, p=.99			0.81 (0.64, 1.01)
**Europe**			
Bhatt, 2021b	NR / 399	NR / 401	0.69 (0.50, 0.95)
Cannon, 2020	224 / 3091	123 / 1546	0.91 (0.73, 1.13)
Radholm, 2018	NR / NR	NR / NR	0.74 (0.57, 0.95)
Wiviott, 2019	211 / 3806	237 / 3823	0.90 (0.73, 1.06)
Bhatt, 2021a	NR / 2324	NR / 2322	0.72 (0.57, 0.90)
I-squared=16.2%,p=.31			0.81 (0.72, 0.91)
**Asia**			
Cannon, 2020	26 / 350	13 / 173	0.96 (0.50, 1.88)
			0.96 (0.50, 1.86)
**Australia_New Zealand**			
Cannon, 2020	9 / 115	21306	0.92 (0.31, 2.74)
			0.92 (0.31, 2.74)
**Asia/Pacific**			
Cannon, 2020	35 / 465	18 / 231	0.95 (0.54, 1.67)
Wiviott, 2019	36 / 1093	37 / 1093	0.97 (0.62, 1.58)
I-squared=0.0%, p=.96			0.96 (0.67, 1.38)
**Africa**			
Cannon, 2020	28 / 251	14 / 126	1.00 (0.53, 1.91)
			1.00 (0.53, 1.90)

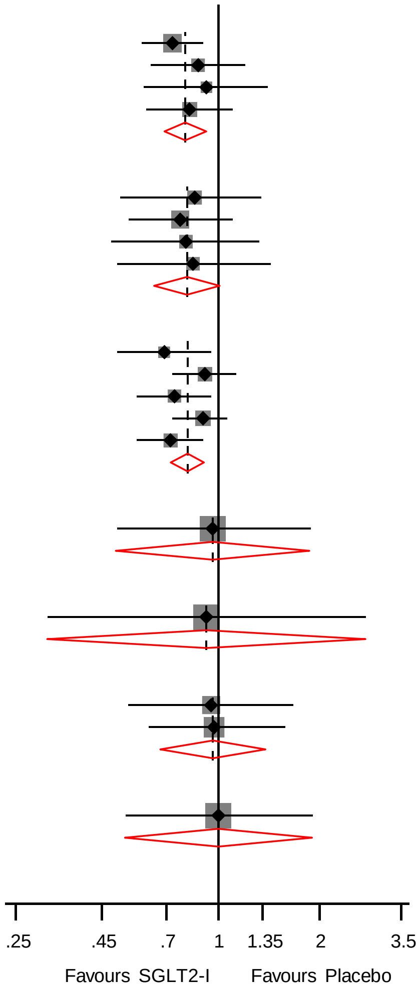

CI, confidence interval (bars); CVD, cardiovascular disease; HF, heart failure; HR, hazard ratio; NR, not reported; SGLT2-I, sodium–glucose co-transporter 2 inhibitor

Appendix 8: Risk for the composite outcome of CVD death or HF hospitalization comparing SGLT2-I with placebo by ethnicity

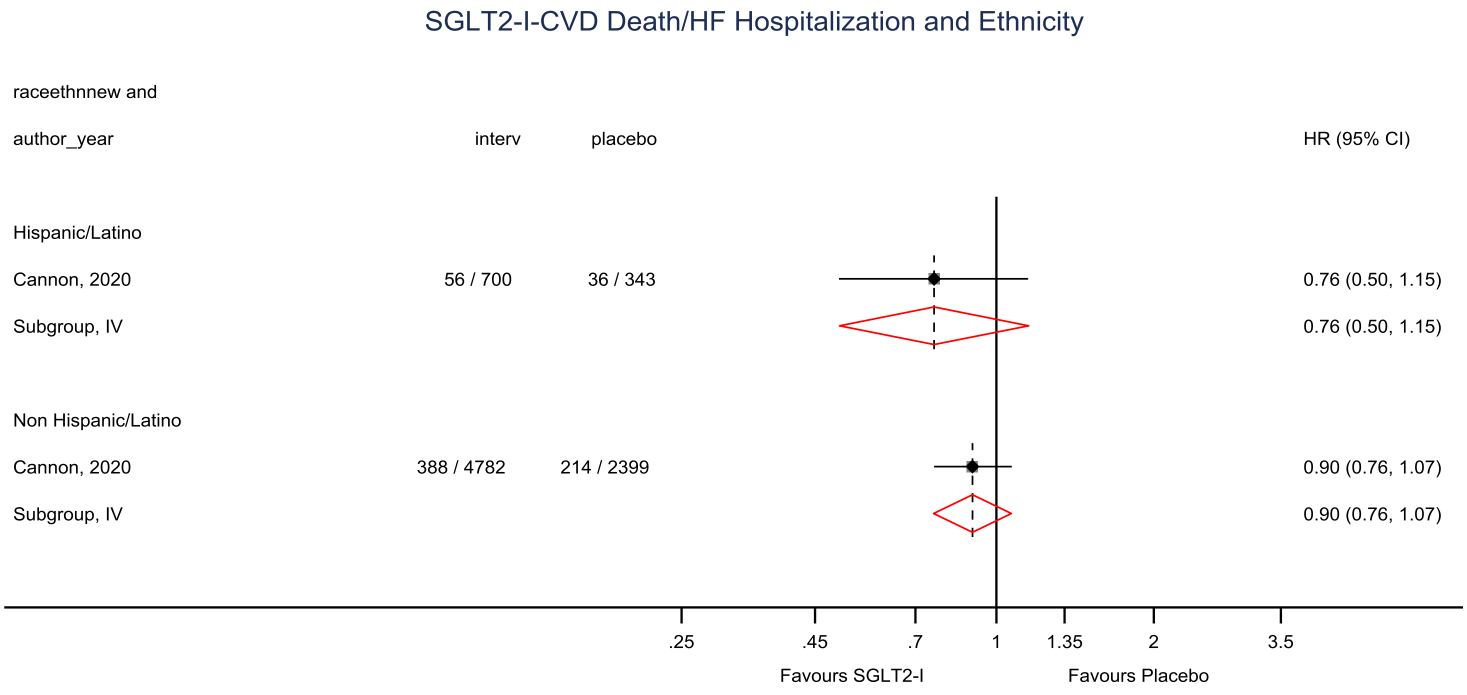

CI, confidence interval (bars); CVD, cardiovascular disease; HF, heart failure; HR, hazard ratio; SGLT2-I, sodium–glucose co-transporter 2 inhibitor

Appendix 9: Risk for the composite outcome of end-stage kidney disease, doubling of creatinine level or death from renal causes comparing SGLT2-I with placebo by region

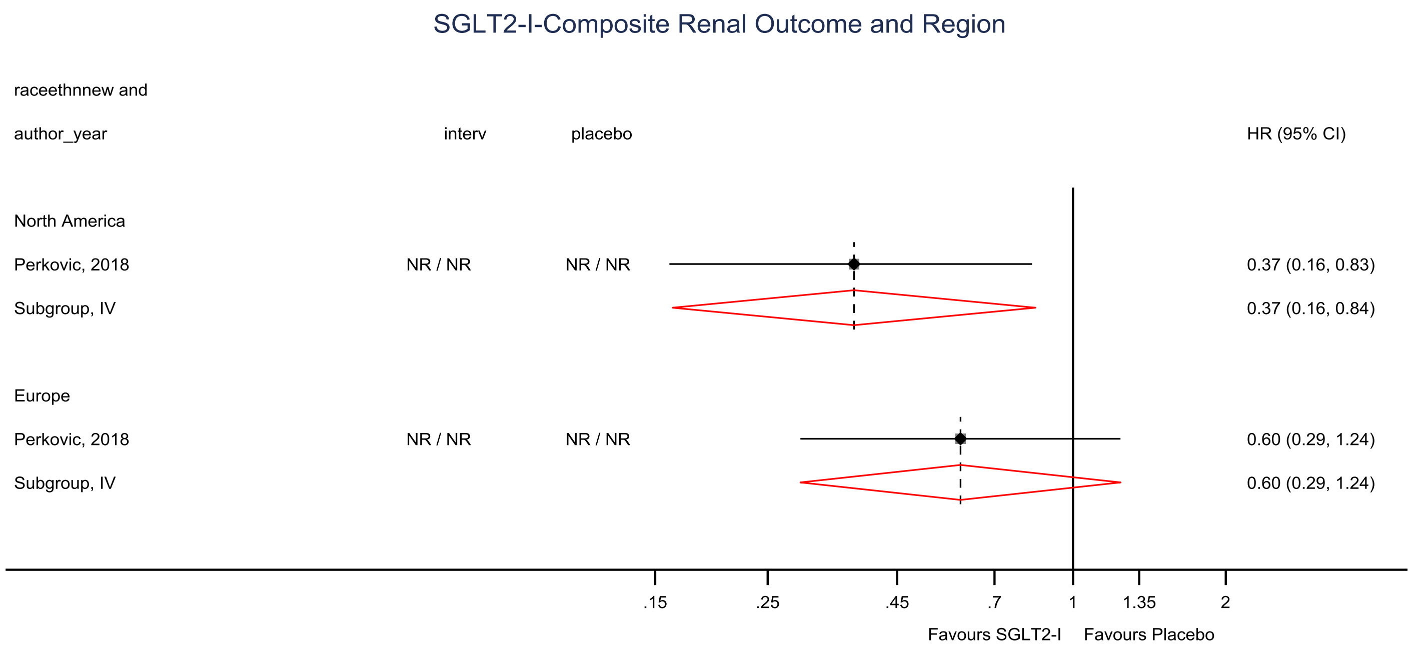

CI, confidence interval (bars); CVD, cardiovascular disease; HR, hazard ratio; NR, not reported; SGLT2-I, sodium–glucose co-transporter 2 inhibitor

Appendix 10: Risk for CVD death comparing SGLT2-I with placebo by race and ethnicity

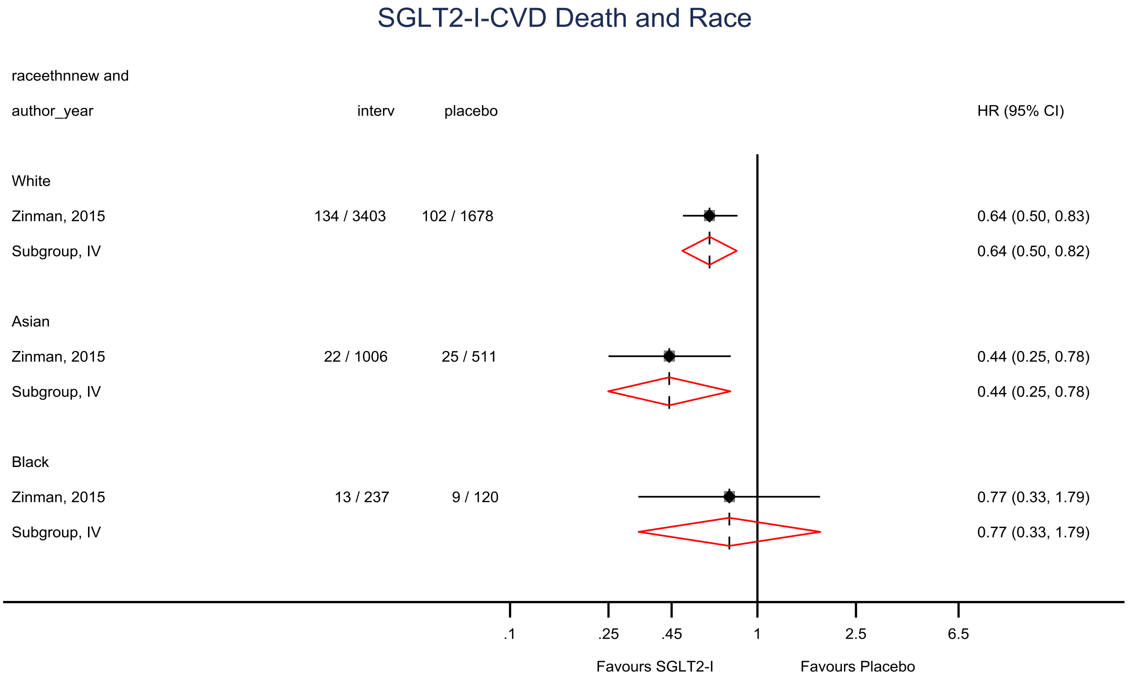

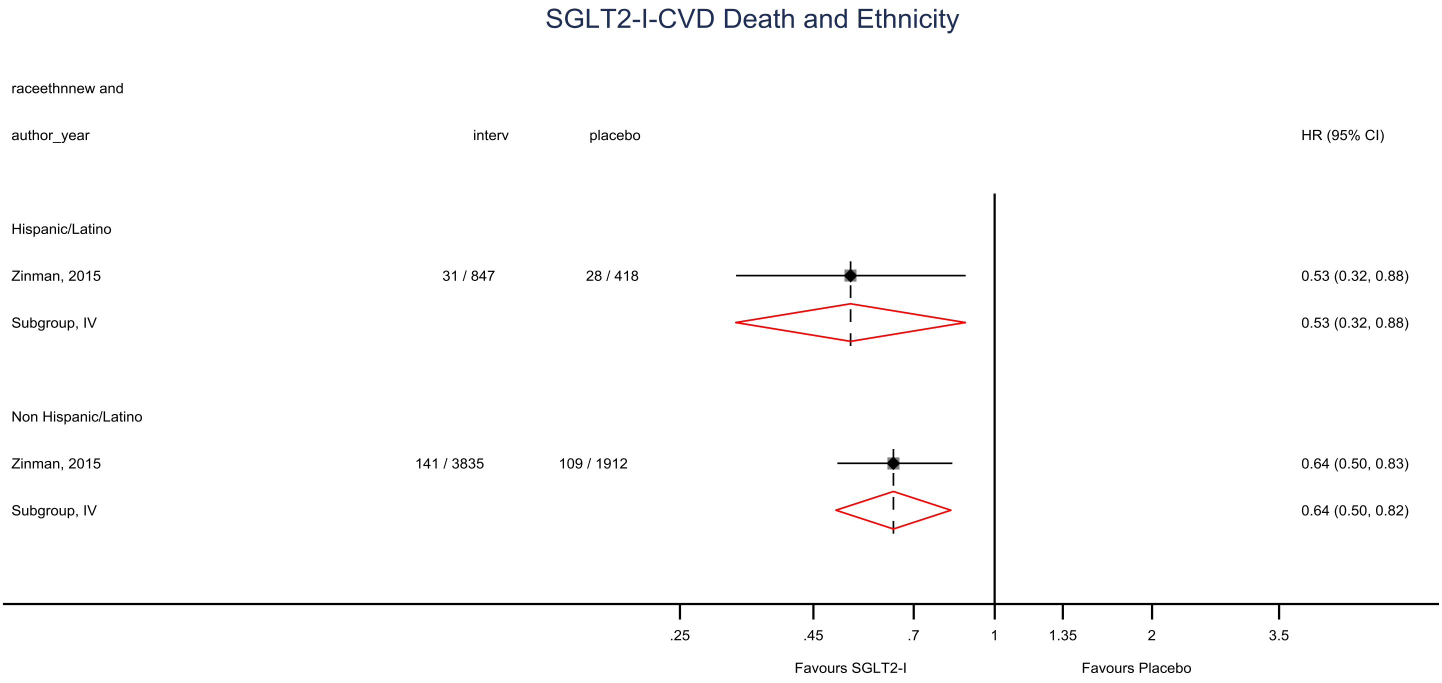

CI, confidence interval (bars); CVD, cardiovascular disease; HR, hazard ratio; SGLT2-I, sodium–glucose co-transporter 2 inhibitor

Appendix 11: Risk for HF hospitalization comparing SGLT2-I with placebo by race and region

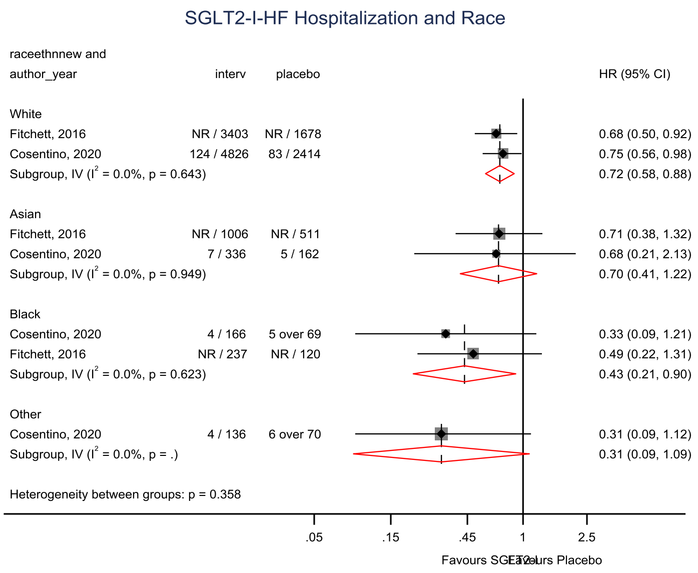

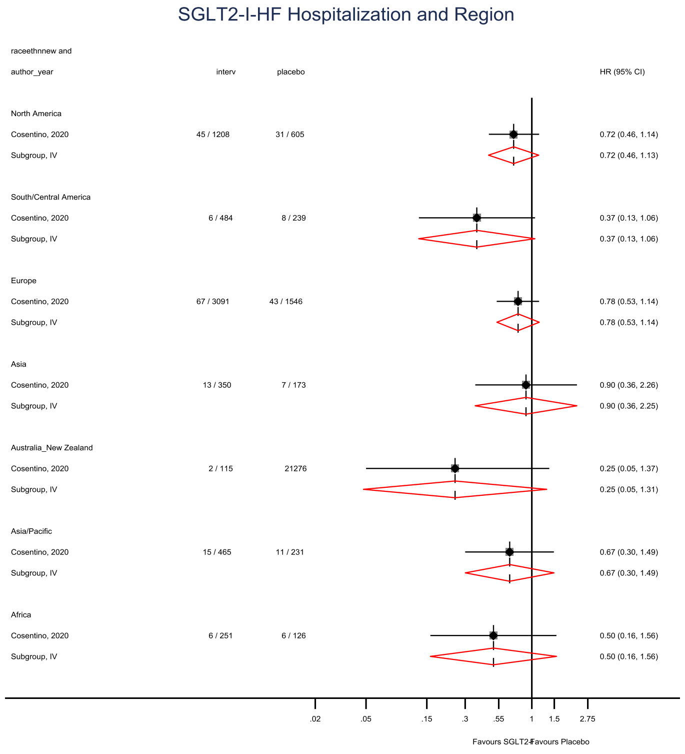

CI, confidence interval (bars); HF, heart failure; HR, hazard ratio; NR, not reported; SGLT2-I, sodium–glucose co-transporter 2 inhibitor

Appendix 12: Risk for incident or worsening nephropathy comparing SGLT2-I with placebo by race

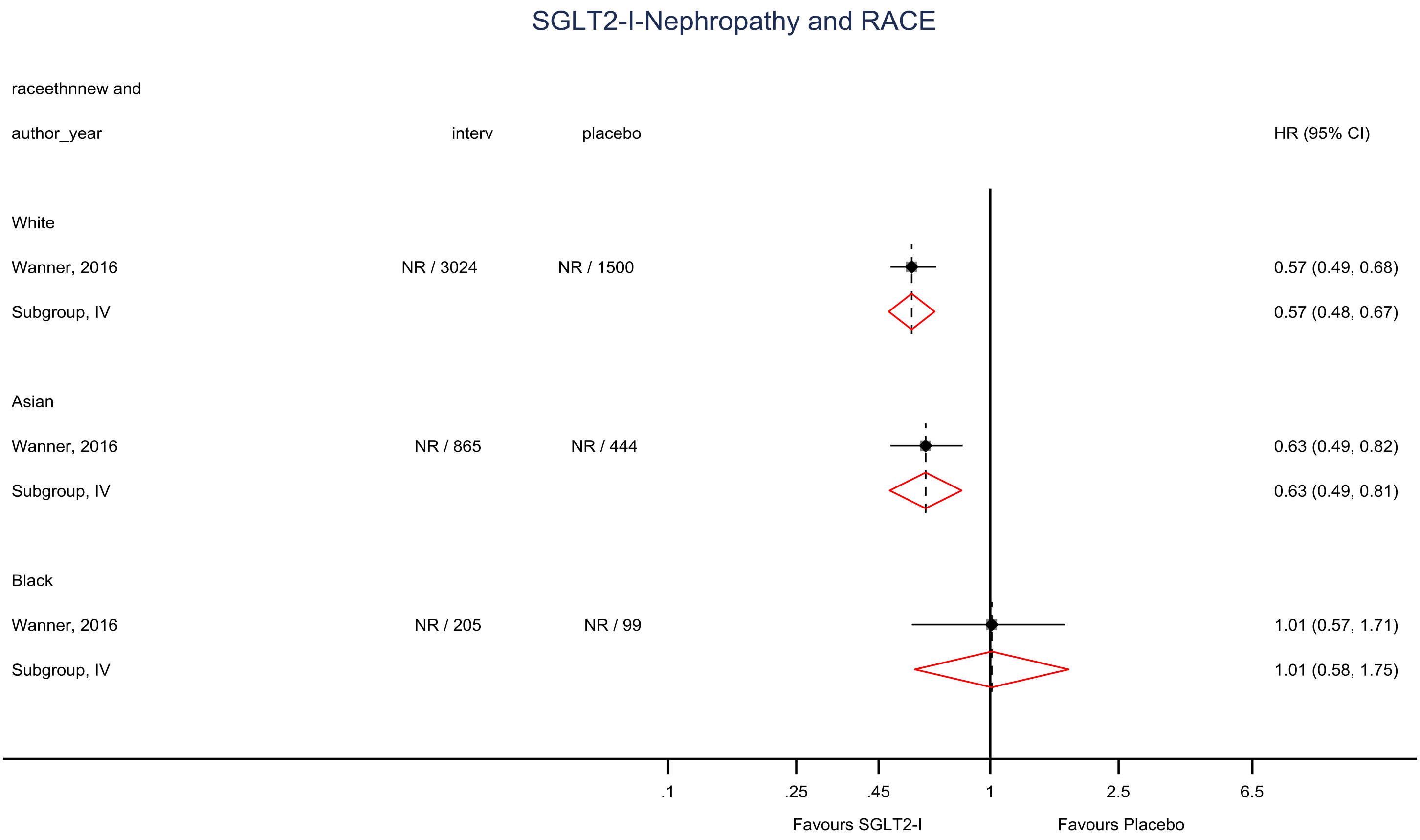

CI, confidence interval (bars); HF, heart failure; HR, hazard ratio; NR, not reported; SGLT2-I, sodium–glucose co-transporter 2 inhibitor

Appendix 13: Risk for the composite outcome of end-stage kidney disease, 40% reduction in eGFR or death from renal causes comparing SGLT2-I with placebo by race and region

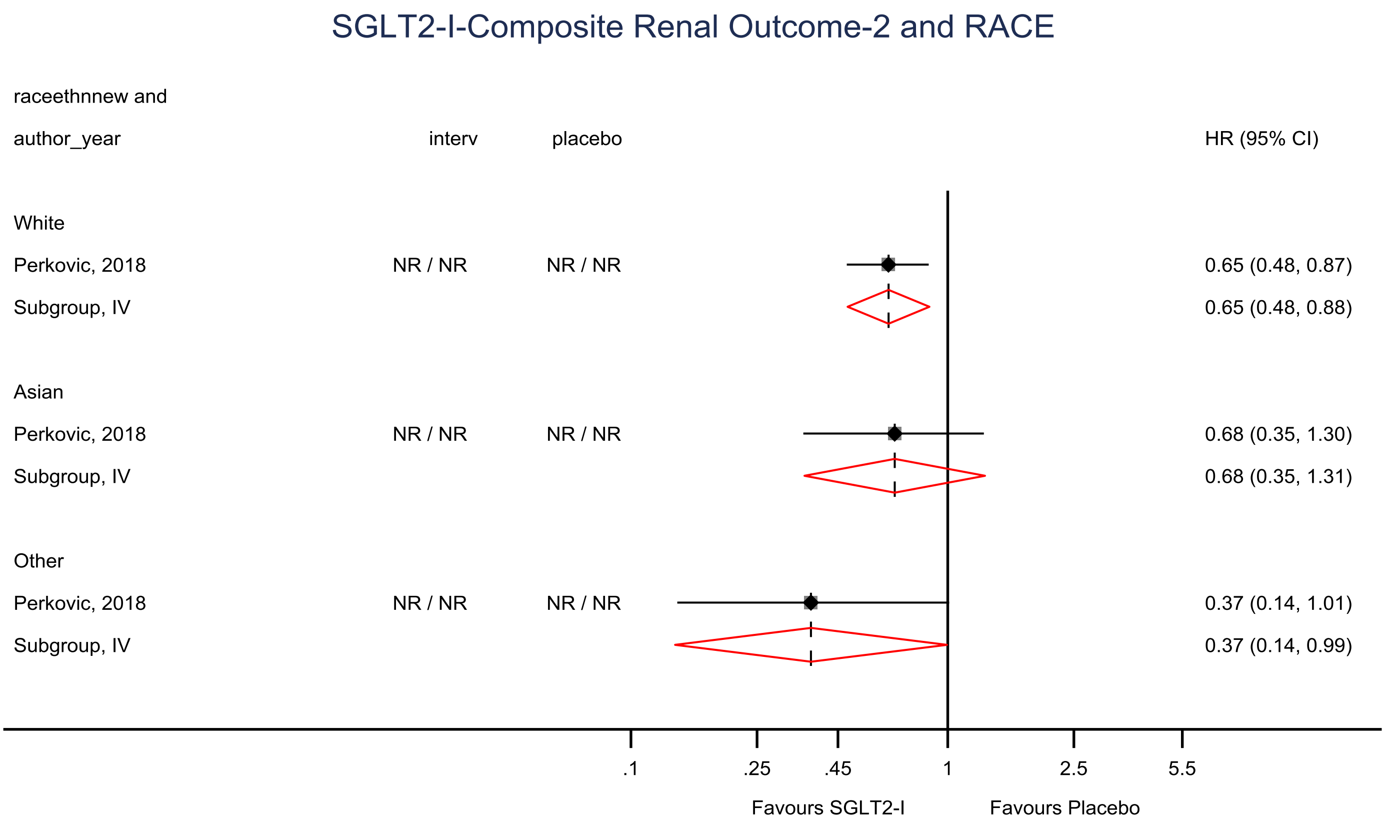

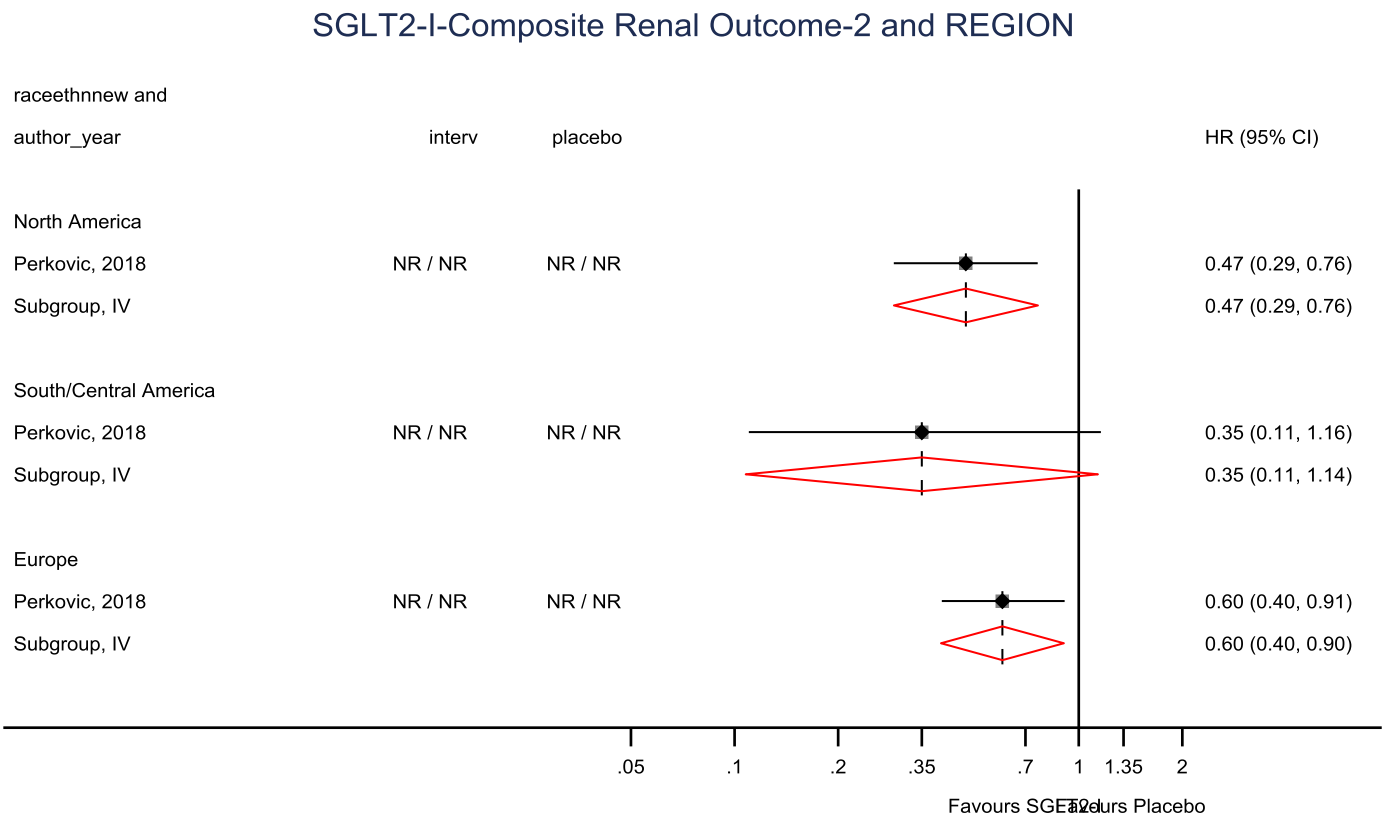

CI, confidence interval (bars); HR, hazard ratio; NR, not reported; SGLT2-I, sodium–glucose co-transporter 2 inhibitor

Appendix 14: Risk for the composite outcome of end-stage kidney disease, doubling of creatinine level or CVD or renal death comparing SGLT2-I with placebo by race and ethnicity

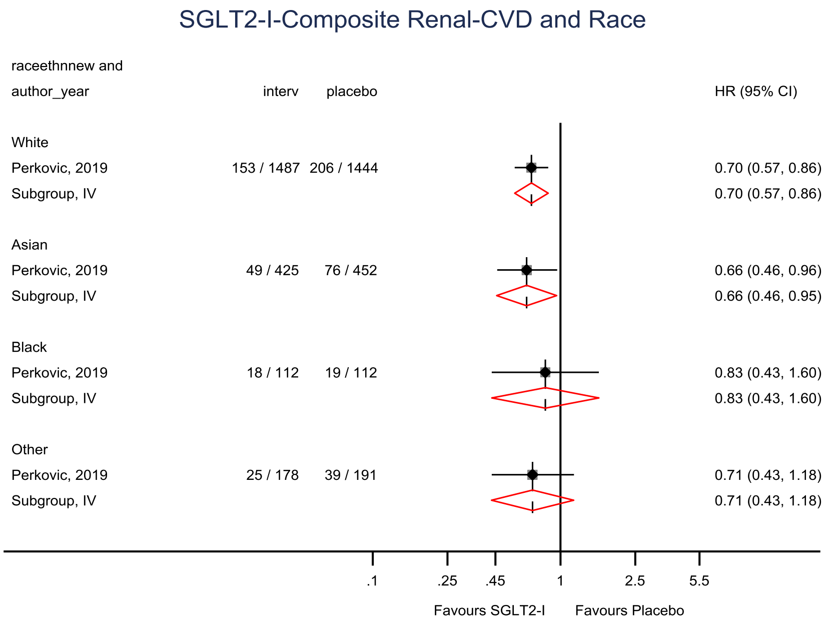

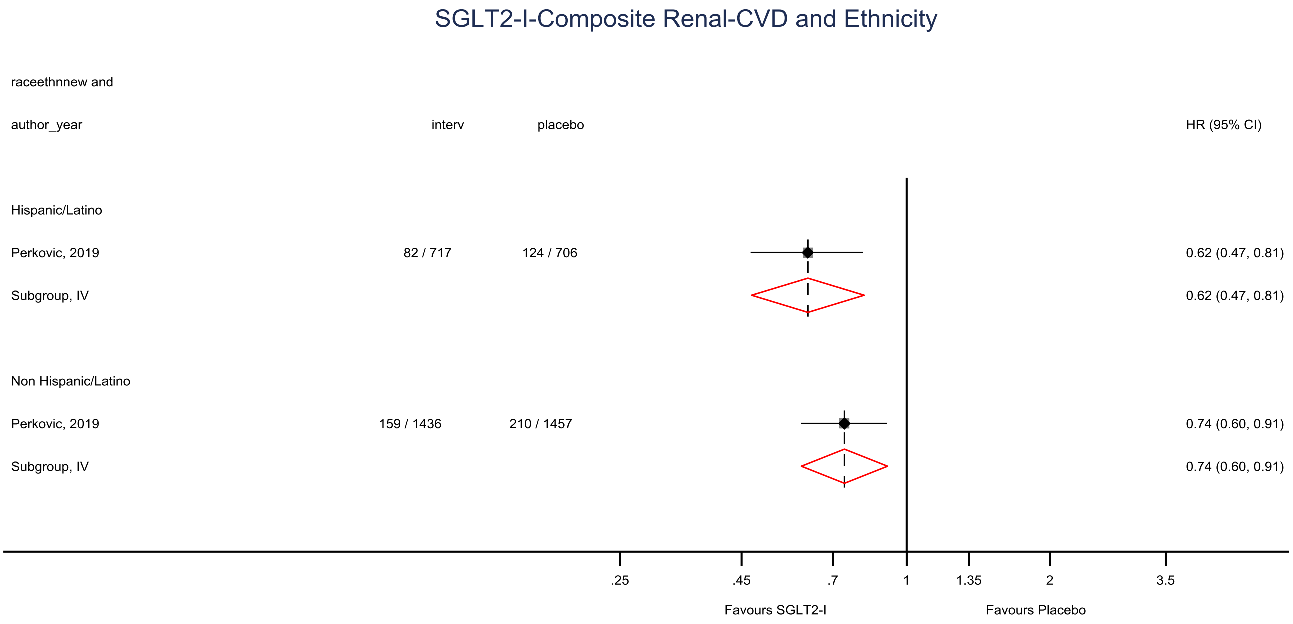

CI, confidence interval (bars); CVD, cardiovascular disease; HR, hazard ratio; SGLT2-I, sodium–glucose co-transporter 2 inhibitor

Appendix 15: GRADE summary of findings

SGLT2-I compared to Placebo in T2D

Outcomes	№ of participants (studies) Follow-up	Certainty of the evidence (GRADE)	Relative effect (95% CI)	Anticipated absolute effects	
				Risk with Placebo	Risk difference with SGLT2-I
MACE in White populations	33911<br>(4 RCTs)	⊕⊕⊕⊕<br>High	**HR 0.92**<br>(0.86 to 0.98)	81 per 1,000	**6 fewer per 1,000**<br>(11 fewer to 2 fewer)
MACE in Asian populations	3298<br>(3 RCTs)	⊕⊕⊕⊕<br>High	**HR 0.69**<br>(0.53 to 0.92)	65 per 1,000	**20 fewer per 1,000**<br>(30 fewer to 5 fewer)
MACE in Black populations	928<br>(3 RCTs)	⊕⊕⊕◯<br>Moderate[a]	**HR 1.11**<br>(0.82 to 1.51)	72 per 1,000	**8 more per 1,000**<br>(13 fewer to 35 more)

Explanations

a. 95% CI ranged from 0.82 to 1.51.

GLP1-RA compared to Placebo for T2D

Outcomes	№ of participants (studies) Follow-up	Certainty of the evidence (GRADE)	Relative effect (95% CI)	Anticipated absolute effects	
				Risk with Placebo	Risk difference with GLP1-RA
MACE in White populations	43340 (7 RCTs)	⊕⊕⊕◯ Moderate[a]	**HR 0.88** (0.80 to 0.97)	110 per 1,000	**12 fewer per 1,000** (21 fewer to 3 fewer)
MACE in Asian populations	4603 (7 RCTs)	⊕⊕⊕⊕ High	**HR 0.76** (0.63 to 0.93)	89 per 1,000	**21 fewer per 1,000** (32 fewer to 6 fewer)
MACE in Black populations	3142 (7 RCTs)	⊕⊕⊕⊕ High	**HR 0.88** (0.68 to 1.16)	121 per 1,000	**14 fewer per 1,000** (37 fewer to 18 more)

*__The risk in the intervention group__ (and its 95% confidence interval) is based on the assumed risk in the comparison group and the **relative effect** of the intervention (and its 95% CI).

CI: confidence interval; **HR:** hazard Ratio

GRADE Working Group grades of evidence
High certainty: we are very confident that the true effect lies close to that of the estimate of the effect.
Moderate certainty: we are moderately confident in the effect estimate: the true effect is likely to be close to the estimate of the effect, but there is a possibility that it is substantially different.
Low certainty: our confidence in the effect estimate is limited: the true effect may be substantially different from the estimate of the effect.
Very low certainty: we have very little confidence in the effect estimate: the true effect is likely to be substantially different from the estimate of effect.

Explanations

a. I-squared value of 62.9%
